# Antimicrobial resistance- and pathogen patterns in the fecal microbiota of sows and their offspring in German commercial pig farms

**DOI:** 10.1371/journal.pone.0290554

**Published:** 2023-08-24

**Authors:** Anja Lührmann, Andrea Palmini, Justinus Hellmich, Vitaly Belik, Jürgen Zentek, Wilfried Vahjen

**Affiliations:** 1 Institute of Animal Nutrition, Freie Universität Berlin, Berlin, Germany; 2 System Modeling Group, Institute of Veterinary Epidemiology and Biostatistics, Freie Universität Berlin, Berlin, Germany; University of Tripoli, LIBYA

## Abstract

Reducing antibiotic use is one of the biggest challenges in pig farming, as antibiotics have been used for years to control typical problems such as newborn or post-weaning diarrhea. The pressure a one health approach has created on animal production regarding antimicrobial resistance is an opportunity to find other strategies against enterobacterial pathogens in suckling and weaned piglets. A farm-specific approach could have a good success due to the individual farm structures in Germany and other countries. In this study, non-metric multidimensional scaling, hierarchical clustering, and latent class analysis were used to determine the impact of antibiotic use on antibiotic resistance patterns and pathogen prevalence in 20 German pig farms. This may help to develop individualized health strategies. 802 fresh fecal samples were collected from sows and piglets from 20 piglet production and rearing farms at different production times (sows antepartum and postpartum, suckling piglets, weaned piglets). In addition, the use of antibiotics was recorded. DNA extracts were subjected to quantitative real-time qPCR with primers specific for antibiotic resistance genes (*int1*, *sul1-3*, *dfrA1*, *mcr-1*, *bla*_*CTX-M*)_, and virulence factors of relevant bacteria (*C*. *difficile*, *C*. *perfringens*, *Salmonella*, *Escherichia*/*Shigella*/*Hafnia*, *E*. *coli*). Linear and logistic regression models were used to analyze the relationship between different antibiotics and the major genes contributing to the clustering of observations for the different animal groups. Clustering revealed different farm clusters for sows, suckling piglets, and weaned piglets, with the most remarkable diversity in antibiotic use among weaned piglets. Amoxicillin, lincomycin, and enrofloxacin were identified as the most probable cause of increased odds of the presence of relevant antibiotic resistance genes (*mcr1*, *dfrA1*, *bla*_*CTX-M*_). Still, direct effects of a specific antibiotic on its associated resistance gene were rare. Enrofloxacin and florfenicol favored the occurrence of *C*. *difficile* in sows. The *E*. *coli* fimbriae genes were less affected by antibiotic use in sows and piglets, but the F4 fimbriae gene could be associated with the integrase 1 gene in piglets. The results confirm that multidrug-resistant enterobacteria are widespread in German pig farms and give awareness of the impact of current antibiotic use while searching for alternative health strategies.

## Introduction

In modern pig production, two problems are facing each other: antibiotic resistance and pathogenic agents, which hinder a desired reduction of antibiotic consumption. Especially newborn and weaning animals are susceptible to diarrheal diseases, which can often only be controlled with antibiotics. Therefore, most antibiotics are used at a young age, especially during the weaning transition, and are usually administered for metaphylaxis [1].

Post-weaning diarrhea (PWD) is mainly governed by enterotoxigenic *Escherichia coli* (ETEC), with fimbriae F4 and F18 being the major pathogenicity factors found in pigs [2]. Environmental factors can be crucial cofactors in this critical phase by affecting gut development, digestive physiology, microbiota, and immune function [3]. Nevertheless, there has yet to be a strategy to prevent this disease completely.

Colistin is widely used for antibiotic control of intestinal disorders in piglets [1]. Still, due to its classification as polymyxin, it is considered one of the critically important antibiotics (CIA) for human medicine by the World Health Organization (WHO). It should therefore be used with prudence [4]. Plasmid-mediated colistin resistance via the *mcr-1* gene, first discovered in China [5], is also prevalent in Europe [6–8] and the link between the occurrence of colistin resistance with the administration of colistin in pig production could be drawn quickly [9].

Another problem are ESBL-producing enterobacteria, which are resistant to beta-lactam antibiotics such as penicillin, amoxicillin, and cephalosporins. For a long time, they have been among the greatest threats to human medicine, and their spread in food-producing animals forms an additional danger of dissemination [10]. In particular, the *CTX-M* types are widely distributed in farm animals, such as pigs and poultry [11,12]. Beta-lactam antibiotics, especially penicillin, and cephalosporins, are used for diseases of various organ systems in every production period in pigs [1]. The 4th and 5th generations of cephalosporins belong, as well as colistin, to the CIA [4]. Other antibiotics used in pig production are tetracyclines, macrolides, and trimethoprim in combination with sulfonamides, fluoroquinolones, aminoglycosides, lincosamides, and pleuromutilins [1].

Mechanisms of resistance transfer have been well studied *in vitro*. Mobile genetic elements on plasmids lead to the easy spread of resistance genes within the bacterial community, and antibiotics enhance this via selection pressure [13]. So-called integrons, which carry antimicrobial resistance genes, play a crucial role in this process, especially for gram-negative bacteria [14]. Some observations also indicate a connection between integrons and virulence factors of *E*. *coli* [15]. Links between antibiotic resistance and virulence could also be described [16], but evidence about the relation between antibiotic use and virulence genes or facultatively pathogenic bacteria is rare.

In Germany, antibiotics dispensed by veterinarians to farmers are documented and monitored by law (German Veterinary Medicine Act). If limits are exceeded, the veterinarian and farmer must initiate measures to reduce them. Since the recording of the quantities of antibiotics dispensed to veterinarians in 2011, there has been a significant decrease in antibiotic use, also in the area of pig production [17]. Nevertheless, not all farms can manage without antibiotics, especially in the critical weaning phase. Because of differences in antibiotic use routines and laws in each country, regional studies are needed before intervention strategies can be developed. Addressing the specific situation of the farm could lead to a change in thinking and help to break management patterns regarding non-indicated antibiotic use [18].

In newborn piglets, besides *E*. *coli*, *Clostridium perfringens* and *Clostridioides difficile* are associated with diarrhea [19]. However, as with PWD, factors other than growth of virulent bacteria appear important in clinical outbreaks of neonatal diarrhea in piglets [20]. In addition, *Salmonella* plays a role throughout pig production. Infections also lead to enteritis, and some *Salmonella* strains can carry a broad spectrum of antibiotic resistance. Even without a clinical outbreak in the host, they pose a risk to the end consumer because pork is one of the most significant vectors for *Salmonella* [21,22].

The present study is part of a larger project called OptiBiom, which aims to combat typical problems in pig production such as neonatal and post weaning diarrhea. An earlier study has already found evidence that there are farm-specific- and sow-piglet-specific patterns in the fecal microbiota composition in German sow farms [23]. Thus, the present study aims to link farm-specific antibiotic use with the occurrence of critical bacteria, virulence factors and antibiotic-resistance genes. To study this relationship in detail, clusters of farms were first characterized based on differences in antibiotic use. Then, the major genes involved were identified and associated with antibiotics using different regression models for the presence/absence and concentration of target genes to assess the potential importance of the antibiotics that contribute the most.

This study focuses on investigating 20 German pig farms for antibiotic resistance genes, bacterial genes, and virulence genes as well as antibiotic treatment at different production times. This approach may help to build knowledge and databases for relevant antimicrobial resistance and virulence patterns in German pig farms. Additionally, the data of the present study might alone help to give awareness to combat routine antibiotic treatment.

## Materials and methods

### Ethics statement

The present study is not an animal experiment as defined by the German Animal Welfare Act (TierSchG 2006). Fecal samples were collected non-invasively. The farm owners gave their written consent to sampling and data collection on their farms and to the publication of our results.

### Study design and sampling

The study design was based on sampling the same sows and their offspring over a period of seven weeks during the production cycle (sows 15 ± 3d antepartum and 11 ± 3 d postpartum, piglets at the age of 11±3 d and 34±3 d). A total of 802 fresh fecal samples were acquired from 10 ± 1 sows and 10 ± 1 piglets from 20 different piglet production and rearing farms. The antibiotic use on the farms was recorded throughout the sampling phase (S39 Table in S1 File). Further details were previously described [23].

### Quantitative real-time PCR

DNA concentration was determined using Promega QuantiFluor^®^ dsDNA System (Promega Corporation, Madison, USA) according to the manufacturer’s instructions. Fluorescence was measured with a microplate reader (Infinite^®^ 200 PRO, Tecan Group AG, Männedorf, Switzerland) at 506 nm_Ex_/535 nm_Em._

DNA extracts were subjected to quantitative real-time qPCR with primers specific for antibiotic resistance genes, pathogen genes, and virulence factors of relevant bacteria (S1 Table in S1 File). Target genes for antibiotic resistance were chosen according to the most used antibiotics on the examined farms. The qPCR was performed with a commercial cycler (Mx3000P; Agilent Technologies Inc., Santa Clara, USA). Each protocol included 40 cycles with an initial activation at 95°C for 10 min, annealing at the given temperatures, and an extension at 72°C for 1 min. Denaturation and final extension were each performed at 95°C for 30 seconds.

### Quantitative analysis of detected target genes

Due to the non-normal distribution of the data, the Mann–Whitney test was chosen to compare the quantitative enumeration of target genes for sow and piglet data and the Kruskal-Wallis test for the comparison of farms. Since the detection of genes was very variable between animal groups, a prevalence analysis was added to compare more clearly. The prevalence between different groups was analyzed with the Chi-Squared test. Statistical procedures were performed using the IBM SPSS Statistics software Version 27 (IBM, Chicago, USA). A level of 95% confidence was deemed as significantly different.

### Nonmetric multidimensional scaling (NMDS) and Hierarchical clustering

Due to a large amount of information on antibiotic use present in the data, a nonmetric multidimensional scaling (NMDS) procedure was used to reduce the dimensionality and improve the visualization of the results. This procedure assesses similarities between samples when considering multiple variables of interest. It creates a matrix of similarities (dissimilarities) for each animal group’s binary variable–application or no application–of different antibiotics. The Jaccard distance was used as a dissimilarity measure, given the dataset structure. Unlike principal component analysis, this procedure was more suitable for the data type at hand since it did not require to fulfill multivariate normality nor multivariate homoscedasticity assumptions. The generated NMDS matrices allowed a hierarchical clustering for the observations in each animal group.

### Latent class analyses

To analyze the presence/absence (binary data) of target genes, the Latent Class Analysis (LCA) was implemented to cluster the data. This method allows the creation of classes or clusters characterized by a pattern of conditional probabilities indicating the chance that a target gene has to take on specific values, also giving probabilities of observations belonging to the class. The LCA was conducted first on a different number of classes to find the optimal number of classes to use for the analysis by comparing the Akaike Information Criterion (AIC) of each model. This optimal number was the same for all animal groups analyzed. Then the procedure was applied repeatedly several times to ensure model optimality. The analysis was conducted separately for the two classes of target pathogenic and resistance genes for both sows and piglets, divided into antepartum and postpartum sows and suckling and weaning piglets. NMDS analysis and LCA were conducted using software R (version 4.1.0.).

### Regression models

Following the LCA on the antibiotic resistance genes, a variable selection procedure has been conducted to detect the most relevant genes contributing to clustering the observations for the different animal groups. Once those genes had been detected, the aim was to analyze their relationship with different antibiotics. A generalized linear regression model has been implemented for those cases with low zeros. In the case of inflation of zeros, first, a logistic model was implemented using the presence/absence of the gene information (1/0 variable) as the dependent variable and then applying the normal linear model on those observations having a concentration greater than zero. This methodology was used to spot the potential differences between those units not having/having a concentration of one of the relevant genes. For those genes where the Shapiro test indicated a poor normal fit, a transformation was applied to the distribution of the gene concentration, scaling it between 0 and 1. Then a beta regression was used in those cases. The application/not application of antibiotics was used as a predictor for the models. In each model, all antibiotics available for that particular animal group were used at the first step, then using a variable selection method based on the AIC indicator. Each model was updated, dropping those predictors (antibiotics), resulting in not being relevant for the model.

## Results

### Quantitative comparison of antibiotic resistance genes, enterobacterial genes, and virulence genes in German pig farms

A total of 406 sow and 396 piglet samples from different production time points and farms were analyzed by quantitative real-time PCR. Prevalence and concentration of enterobacterial antibiotic resistance or related genes (*bla*_*CTX-M*_, *sul1-3*, *dfrA1*, *mcr-1*, *int1*), bacteria, and virulence genes (*C*. *perfringens* (*cpa*), *C*. *difficile* (*Cdiff*)), *Salmonella* (*QVR*), *Escherichia/Hafnia/Shigella* (*Escherichia*), and *E*. *coli* virulence factors (*fae*, *fedA*, *fas*) were recorded. The whole data set is shown in the supporting information (S2-S5 Tables in S1 File).

Except for Salmonella, significant differences were observed between farms for the concentrations of all other analyzed genes (S2 Table in S1 File). Highly prevalent in all farms were the antibiotic resistance-related genes *int1* (100%) and *sul1-3* (>98%), as well as the 16S rDNA copy numbers for the *Escherichia* group (>98%) (S2 Table in S1 File). The lowest prevalence was detected for *Salmonella* (6%) and the *mcr-1* gene for colistin resistance (26%). Overall, *E*. *coli* fimbriae genes were also seen at low prevalence (<20%). Genes for *C*. *perfringens* and *C*. *difficile*, as well as for trimethoprim resistance (*dfrA1*) were highly prevalent (>76%), while the gene for ESBL (*bla*_*CTX-M*_) had a wide range (27–78%) of prevalence between farms (S3 Table in S1 File).

The comparison of antepartum and postpartum sows showed that *C*. *perfringens* and *C*. *difficile* were of significantly higher prevalence in postpartum sows, but concentrations were not affected (S4 Table in S1 File). Generally, all studied genes showed higher concentrations in postpartum sows, except for the *int1* gene. However, the prevalence for the *E*. *coli fedA* gene, trimethoprim- (*dfrA1*), and colistin (*mcr-1*) resistance significantly decreased after birth, while the prevalence for the ESBL gene *bla*_*CTX-M*_ significantly increased.

Suckling and weaned piglets showed significantly lower concentrations and the prevalence of *C*. *perfringens* and *C*. *difficile* in weaned piglets, while only a marginal, albeit significant, increase for the *Escherichia* group was observed (S5 Table in S1 File). All antibiotic resistance genes except trimethoprim resistance (*dfrA1*) and the related enterobacterial integrase I showed significantly lower concentrations in weaned piglets. Similarly, the prevalence of these genes also significantly decreased after weaning. Regarding the *E*. *coli* fimbriae genes, the *fedA* gene displayed a drastic increase in both quantity and prevalence. The prevalence of the *fae* gene increased after weaning, while the *fas* gene decreased significantly.

### Farm-specific distribution of antibiotic use

**Fig 1:**
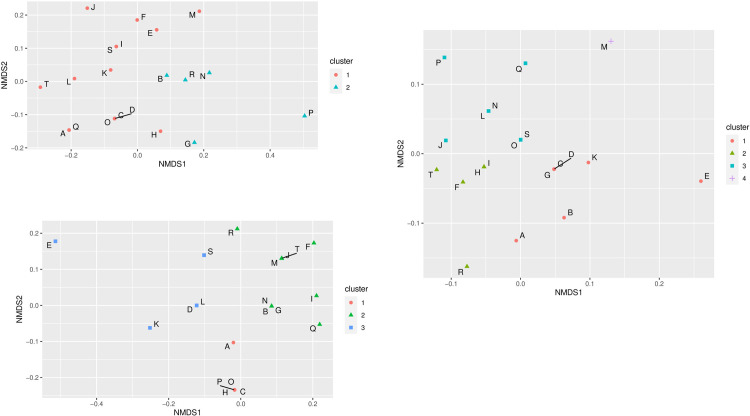
Non-metric multidimensional scaling and hierarchical clustering of antibiotic use for sows (A), suckling- (B), and weaned piglets (C) in 20 German pig farms (A-T).

A non-metric multidimensional scaling (NMDS) approach with a hierarchical clustering analysis was employed to differentiate individual farms according to their antibiotic use to investigate possible farm-specific patterns. First, the NMDS analysis found farms that belonged to the same similarity cluster. Due to the use of different antibiotics in sows and piglets, different clusters formed. Thus, two NMDS clusters were found for sows (Fig 1A). The larger of the two clusters contained 15 farms, while the second cluster was smaller, with only five farms (B, G, N, P, R). This cluster was formed mainly by using amoxicillin, enrofloxacin, and lincomycin, which are much less or absent in cluster 1. For suckling piglets, cefquinome and ceftiofur were used mainly in cluster 1 (5 farms), while the largest cluster (10 farms) was formed primarily by the use of ceftiofur and amoxicillin (Fig 1B). The third cluster was most heterogeneous in terms of antibiotic use, being the only cluster using florfenicol, gentamycin, oxytetracycline, trimethoprim, ceftiofur, and streptomycin. For weaned piglets, also three main clusters emerged with an additional cluster that contained only farm M (Fig 1C). However, compared to the clustering of farms in suckling piglets, the farm identity in the clusters of weaned piglets was different except for cluster 2. Here, all farms of the weaned piglet cluster can be found in the large cluster 2 of suckling piglets.

### Prevalence of antibiotic resistance genes, enterobacterial genes, and virulence genes in antibiotic-use clusters of sows and piglets

**Fig 2:**
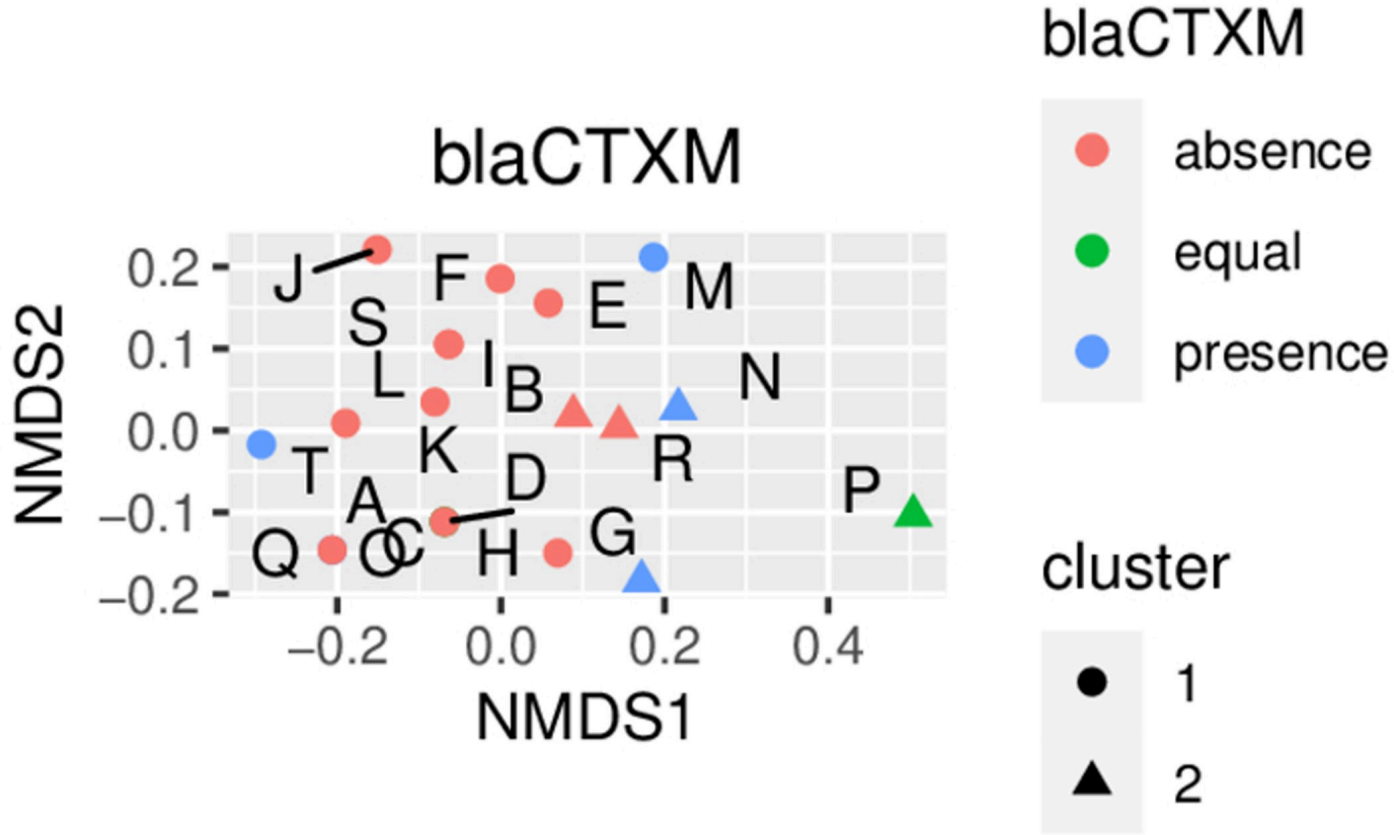
Example for discriminating antibiotic use cluster and presence of the *bla*_*CTX-M*_ gene in postpartum sow samples from 20 German pig farms.

After identifying distinct farm clusters for the different animal types, the prevalence of antibiotic resistance genes, bacterial pathogens, and *E*. *coli* fimbriae was analyzed by cluster. For example, a higher prevalence of *bla*_*CTX-M*_ -positive farms was calculated for postpartum sows from farms belonging to cluster 2 (Fig 2).

This analysis was completed for all measured factors, and associations with farm clusters were established. Due to a large number of plots, Table 1 shows the summary of observations from the combination of NMDS analysis with hierarchical clustering and the prevalence of all measured genes. Exact distributions of all genes are presented in the supporting information (S1-S8 Figs in S1 File).

**Table 1:** Summary of the prevalence of presence (+) and absence (-) of relevant genes in clusters of sows and piglets clustered by antibiotic use.

Gene	Sows		Piglets	
	Antepartum	Postpartum	Suckling	Weaned
*bla*_*CTX-M*_	-	+ Cluster 2	+	- Cluster 2; 3
*mcr1*	-	-	- Cluster 2	-
*C*. *difficile*	+ Cluster 2	+	+	+ Cluster 2
*C*. *perfringens*	equal +/-	+	-	+
*Escherichia*			+	
*fae*				+ Cluster 4
*fas*	+ Cluster 1	+Cluster 2		
*fedA*	equal +/-	-	-	+ Cluster 1

+/- = prevalence of presence/absence in all clusters/farms.

The gene prevalence showed that some genes could be assigned to specific antibiotic use clusters depending on the animal group. No associations were found for *dfrA1* and *Salmonella*.

There is a general absence of *bla*_*CTX-M*_ in antepartum sows in both clusters. However, an increased prevalence of *bla*_*CTX-M*_ could be assigned to postpartum sows, cluster 2. Further differences between sow groups were observed for clostridial genes and *E*. *coli* fimbriae (S3 and S4 Figs in S1 File). In antepartum sows, the absence of *C*. *difficile* is predominant in cluster 1, while in postpartum sows, it is predominantly absent in both clusters. The presence/absence of *C*. *perfringens* and *fedA* is almost equally distributed in antepartum sows. Still, in postpartum sows, *C*. *perfringens* is mainly present, and *fedA* is primarily absent in all farms. In antepartum sows, a prevalence of presence in cluster 1 was observed for *fas*, while it was assigned to cluster 2 in postpartum sows.

Only the *mcr-1* gene could be assigned to a distinct cluster (cluster 2) in suckling piglets. In post-weaning piglets, there were more significant differences in gene prevalence between clusters. While *bla*_*CTX-M*_ is present in suckling piglets in all farms, it is absent in post-weaning piglets, especially in clusters 2 and 3. In addition, *C*. *difficile* is mainly present in cluster 2, while it is equally distributed in cluster 1. The *cpa* gene is more prevalent in all farms, in contrast to the suckling piglets. *FedA* is predominantly absent in all farms, as in the suckling piglets, but it is more abundant in cluster 1. *Fae* is present in cluster 4 (only one farm) but similar to suckling piglets, absent in all other clusters.

### Association of antibiotic use with the most relevant antibiotic resistance genes

Finally, the Latent Class Analysis (LCA) enabled us to select the most relevant genes that contribute to the clustering of observations for the different animal groups. The LCA analysis showed that the antibiotic resistance genes for trimethoprim (*dfrA1*), colistin (*mcr-1*), and the ESBL gene *bla*_*CTX-M*_ were most relevant for clustering. However, time- and animal-related differences were noticed. Thus, *dfrA1* and *bla*_*CTX-M*_ were most relevant in ante- and postpartum sows, while in suckling piglets *dfrA1* and *mcr-1* were most relevant. All three antibiotic resistance genes were relevant in weaned piglets. The association of relevant antibiotic resistance genes with individual antibiotics use on the farms was studied depending on the data via general linear or beta linear regression on gene concentration or logistic regression on the odds of presence (S6-S20 Tables in S1 File). The logistic model gives us a factor by which the odds of the presence of a resistance gene is increased when using a certain antibiotic as against not using the antibiotic. Thus, the odds of the presence of the trimethoprim resistance gene significantly increase when amoxicillin is used in ante- and postpartum sows (Table 2). In the linear model, the most significant antibiotic for antepartum sows was found to be lincomycin, where sows receiving it have a mean concentration 0.3 times less than the sows not receiving it (S7 Table in S1 File). Regarding Amoxicillin application, the same trend for *dfrA1* presence or concentration was visible in piglets. Significant relations between the trimethoprim resistance gene *dfrA1* and trimethoprim application were only found in postpartum sows (Table 2). In addition, in postpartum sows, the presence of trimethoprim resistance seemed to be affected by more antibiotics than in other animal groups. In suckling piglets, the use of amoxicillin and penicillin G increased the odds of the presence of the *mcr-1* gene (Table 2). In weaned piglets, the odds of the presence of colistin resistance were significantly enhanced by the use of lincomycin, enrofloxacin, and doxycycline, while amoxicillin showed only a slight increase. Gene concentration of *mcr-1* was increased by cefquinome and oxytetracycline (S18 Table in S1 File).

**Table 2:** Significant odds ratios of the presence of resistance genes in sows and piglets treated with certain antibiotics (p≤0.05).

Antibiotic agent	*dfrA1*	*bla*_*CTX-M*_	*mcr-1*	Animal
Amoxicillin	7.42 2.89		9.2	Sows antepartum Suckling piglets
Amoxicillin (oral)	0.06 6.87		0.21	Sows antepartum Sows postpartum Weaner
Cefquinom		3.32		Weaner
Doxycyclin (oral)		0.36	3.35	Weaner
Enrofloxacin	2.81	0.16	4.97	Sows postpartum Weaner
Florfenicol	4.43	3.95		Sows postpartum
Lincomycin		76.67	14.64	Sows postpartum Weaner
Lincomycin (oral)		21.0		Weaner
Neomycin (oral)		17.14		Sows postpartum
Penicillin G*			10.29	Suckling piglets
Trimethoprim**	3.04			Sows postpartum

* in combination with streptomycin;

**in combination with sulfadimidine.

The ESBL resistance gene *bla*_*CTX-M*_ showed only associations with antibiotic use in postpartum sows and weaned piglets (Table 2). Strikingly, the use of lincomycin drastically increased the odds of the presence of *bla*_*CTX-M*_ in both groups. Amoxicillin use, which played a major role in the *dfrA1* and the *mcr-1* gene, was not to be associated with the *bla*_*CTX-M*_ presence.

### Association of individual antibiotics with the most relevant bacteria and virulence factors

Similar to the search for the most relevant antibiotic resistance genes, the computation of the most relevant bacterial pathogens yielded time- and animal-related differences (S21-S38 Tables in S1 File). The variable selection applied to the latent class analysis of bacterial genes for sows showed that *C*. *difficile*, *fedA*, and *fas* were the most relevant genes (Table 3).

**Table 3:** Significant odds ratios of the presence of *C*. *difficile* and *E*. *coli* fimbriae genes *fae* and *fedA* in sows treated with certain antibiotics (p≤0.05).

Antibiotic agent	*C*. *difficile*	*E*. *coli* fas	*E*. *coli* fedA	Sampling time
Amoxicillin		3.04		antepartum
Enrofloxacin		0.32 2.76	3.29	antepartum postpartum
Lincomycin		0.24 0.22	0.22	antepartum postpartum
Neomycin (oral)			0.15	antepartum
Trimethoprim*	0.51	0.31		antepartum

*in combination with sulfadimidine.

Thus, using enrofloxacin and florfenicol significantly increased the concentration of *C*. *difficile* in both antepartum and postpartum sows (S26 Table in S1 File). In contrast, trimethoprim significantly increased the odds of *C*. *difficile* presence only in antepartum sows (Table 3). For postpartum sows, no significance was found for both the logistic and the beta model regarding the *fedA* gene. The reason is the very small sample size of ‘nonzero’ values for both the gene’s presence and positive concentration values. This was the same for the *QVR* gene of *Salmonella*, which was also found to be relevant in the clustering of postpartum sows.

In piglets, *C*. *perfringens*, the *Escherichia/Shigella/Hafnia* group, and *E*. *coli* fimbriae gene *fae* are the most significant genes in clustering. The use of enrofloxacin, amoxicillin, and colistin results in increased odds of the presence of *C*. *perfringens in* post-weaning piglets (Table 4). Notably, Penicillin G and Tulathromycin sharply increased the odds of *C*. *difficile* presence in suckling piglets.

**Table 4:** Significant odds ratios of the presence of *C*. *difficile*, *Escherichia*, and the *E*. *coli* fimbriae genes *fae* and *fedA* in piglets treated with certain antibiotics (p≤0.05).

Antibiotic agent	*C*. *difficile*	*C*. *perfringens*	*Escherichia*	*E*. *coli fae*	*E*. *coli fedA*	Sampling time*
Amoxicillin			5.25	5.34	0.27	Before After
Amoxicillin (oral)		0.42				After
Ceftiofur				0.14		Before
Colistin				5.02		Before
Colistin (oral)		0.30				After
Doxycyclin (oral)				2.66	3.76	After
Enrofloxacin		0.38				After
Penicillin G**	5.20					Before
Tulathromycin	4.98					Before
Gene Integrase 1			3.32	2.56		Before After

*Regarding weaning;

**in combination with streptomycin.

The logistic regression result highlights the importance of the *int1* gene for piglets, which increases the odds of the presence of *Escherichia* in suckling piglets and *fae* in weaned piglets (Table 4). The beta model also found that the concentration of Escherichia and *fae* increased with each unit increase in the *int1* gene (S23 Table in S1 File). The same trend for *Escherichia* was observed with amoxicillin in suckling piglets (S32 Table in S1 File).

The beta regression model also showed some negative associations. Thus, Enrofloxacin and colistin decreased the concentration of *Escherichi*a in suckling piglets (S32 Table in S1 File). The parenteral use of amoxicillin decreased the concentration of *C*. *difficile* in piglets after weaning, whereas the concentration was increased with oral administration (S34 Table in S1 File).

## Discussion

The present study is part of the research project ‘Optibiom’, which combines metadata from farms with data from the fecal microbiota of sows and piglets to find a framework for the development of new health strategies. Thus, this study aimed to associate antibiotic resistance patterns and the presence of (facultative) bacterial pathogens and virulence factors with antibiotic use on 20 German pig farms. For this purpose, we used different methods to find answers to a chain of questions successively. First, we quantified a number of relevant genes via qPCR. As expected, farm-specific differences existed for most antibiotic resistance genes, as well as for *C*. *perfringens* alpha toxin, *C*. *difficile*, and the *Escherichia group*. Interestingly, the integrase I (*int1*) and the sulfonamide (*sul-3*) resistance showed relatively high and much more uniform results among those specific genes. These two genes are more or less ubiquitous in German pig farms. The *sul3* gene was detected 20 years ago and was already present in one-third of the sulfonamide-resistant pathogenic *E*. *coli* isolates from pigs in Switzerland [24]. However, later studies in the United Kingdom identified only 9% *sul3* carrying bacterial isolates from manured soils and pig slurries [24]. In this study, the total amount of *sul1-3* genes was measured and may yield a complete picture of *sul1-3* mediated sulfonamide resistance potential of the fecal resistome in pigs. The presence of *int1* has been associated with increased antibiotic resistance in antibiotic-resistant *E*. *coli* isolates [25]. Thus, the potential for an increased exchange of genetic information should be considered a risk factor. Moreover, the *int1* gene can be closely related to sulfonamide resistance in uropathogenic *E*. *coli* [26], which may explain the ubiquitous presence of *int1* and *sul1-3* in this study. Furthermore, this acknowledges the far-spread potential of enterobacteria to incorporate foreign DNA via integrons. Finally, due to its ubiquitous presence, the *int1* gene may be a suitable quantitative indicator gene to assess the potential for gene transfer in enterobacterial populations in pigs. A higher quantity of the fedA gene was also observed in weaned piglets than in suckling. In contrast, most other pathogens and antibiotic-resistance genes showed comparable or lower quantities after weaning. This may be because pathogenic *E*. *coli* are typical post-weaning pathogens that capitalize on the intestinal disorder caused by the drastic changes in the animal that occurs during weaning [27]. On the other hand, *C*. *difficile* and *C*. *perfringens* are pathogens that primarily infect suckling piglets [19,28]. Thus, their reduced numbers and prevalence may result from the changing environmental conditions under which these pathogens occur.

From quantitative qPCR data, we also calculated the prevalence of the respective genes. The prevalence of the *mcr-1* gene was relatively low compared to the other genes. Nevertheless, it has been found on every farm and is more prevalent than in earlier investigations in Germany [6,29]. In the present study, it was found in 26% of the samples. The data are consistent with a study in Spanish farms from 2019 that also detected the *mcr-1* gene in nearly 30% of samples [30].

Birth led to a quantitative increase of most pathogens and antibiotic resistance genes in sows. This is expected because parturition introduces drastic changes on many levels for the animal and, consequently, bacterial composition changes [31]. However, prevalence data also indicates that this effect was farm-specific, as the overall prevalence of the F18 fimbriae (*fedA*) or the colistin resistance (*mcr-1*) was lower after birth in some farms.

Data on antibiotic use were then submitted to NMDS analysis and hierarchical clustering to detect possible cluster formation on the farms. This revealed the presence of distinct antibiotic-use clusters of farms. Antibiotic use between the three observation times (sows, suckling piglets, weaners) was heterogeneous within the farms, so a different number of clusters resulted for all three groups. Differences with high significance were found in weaner pigs, where 4 clusters were formed. In a study about antibiotic drug usage in German pig farms from 2013 to 2015, twelve antibiotic drug classes were identified used for weaner pigs [32]. Individual treatments in every farm were also common in the farms of the present study. Post-weaning problems may have led to more individual use of different antibiotics since there is no general protocol for antibiotic usage in Germany. Previous data showed that the age of the animals and the veterinarian are main factors influencing antibiotic use in pig farms in Germany [33].

Next, Latent Class Analysis was performed to detect the most relevant antibiotic resistance genes that were associated witrh antibiotic use. However, antibiotic use clusters in sows and piglets yielded only a few significant relations. The exclusive use of certain antibiotics (amoxicillin, enrofloxacin, and lincomycin) in sow cluster 2 did not automatically lead to an increased prevalence of respective antibiotic resistance genes. The missing associations for colistin use and colistin resistance *gene mcr-1*, as well as for cefotaxime use and cefotaxime resistance gene *bla*_*CTX-M*_, also exemplify this. Thus, there is no direct causal relationship between the prevalence of antibiotic resistance genes and antibiotic use. Instead, indirect effects that affect the intestinal microbiota may have come into play. For example, in elderly humans, *C*. *difficile* has been known to colonize the colon after antibiotic treatment [34] because there is less competition from displaced antibiotic-susceptible bacteria [35]. Furthermore, the application of antibiotics can drastically impact the bacterial composition in pigs [36]. Thus, it is conceivable that the association of certain antibiotic-resistance genes with unrelated antibiotics results from profound changes within the intestinal microbiota and possibly the development of multidrug-resistant bacteria.

Finally the LCA- and regression model analysis detected colistin- (*mcr-1*), trimethoprim- (*dfrA1*), and cefotaxime (*bla*_*CTX-M*_) resistance genes as the most relevant factors for cluster formation. Trimethoprim (a folate reductase inhibitor), in combination with sulfonamides, is often used in German sow and piglet production [33]. Only postpartum sows showed significant direct relations between trimethoprim use and the trimethoprim resistance gene *dfrA1*. However, amoxicillin enhanced the odds of *dfrA1* prevalence in antepartum and postpartum sows and weaned piglets. The association between trimethoprim resistance and amoxicillin use has been shown in humans [37]. The authors suggested that “co-selection by these antibiotics is an important driver of trimethoprim resistance …”.

The use of colistin is also quite common in pig production, especially in weaned piglets [1]. Therefore, it is not surprising that no significant associations between colistin use and any antibiotic were found in sows. No significant direct relation between colistin and its resistance gene was found in piglets either. Still, the association of a range of antibiotics to increased odds of colistin resistance in suckling piglets shows that even the suckling piglet could be a reservoir for multidrug-resistant enterobacteria. Especially the use of lincomycin considerably increased the chance of colistin resistance in weaned piglets. Lincomycin also drastically increased the odds for the *bla*_*CTX-M*_ gene that codes for resistance against cefotaxime, which is problematic because extended-spectrum-beta-lactamase (ESBL) producing bacteria have developed into a global threat [10]. Although ESBL bacteria are not pathogenic *per se*, the frequent incorporation of foreign DNA by enterobacteria can produce pathogenic ESBL-carrying bacteria that are difficult to treat. As antibiotic use was only recorded in one production cycle, it is possible that previous antibiotic use and thus resistance profiles have interfered with the present antibiotic use. Regression analysis does not depict causation and thus, this bias cannot be completely ruled out.

Regarding associations of facultatively pathogenic bacteria and their virulence factors to antibiotic use, we found that only enrofloxacin and florfenicol favored the odds of the presence of *C*. *difficile* in sows before and after parturition. *C*. *difficile* infects only newborn piglets; thus, increased odds for *C*. *difficile* in mother sows should be avoided, as maternal contamination with *C*. *difficile* can be carried over to the offspring. However, both antibiotics played no role in suckling- or weaned piglets. Resistance against enrofloxacin and florfenicol is common in *C*. *difficile* strains [38,39], and thus, the replacement of sensitive bacteria may have opened the opportunity for this pathogenic bacterium to colonize the suckling piglet [40]. The *E*. *coli* fimbriae genes *fedA*, *fas*, and *fae* were less affected in sows and piglets by antibiotic use. Like lincomycin, enrofloxacin use impacted the odds of the presence in sows but not in piglets. Farm-specific antibiotic use generally had a lesser effect on the presence of facultative pathogens or virulence factors than on those of antibiotic resistance genes. The reverse conclusion could be that the genetic transfer of antibiotic resistance patterns due to antibiotic pressure does not directly produce antibiotic-resistant pathogens [41]. In *E*. *coli* there are findings of a link between antibiotic resistance and virulence but also less of a relation between antibiotic use and virulence [42]. In the present study the *int 1* gene, for example, could also be associated with the virulence gene *fae* for F4 fimbriae and the *Escherichia* group. The logistic regression showed increased odds of presence with the simultaneous presence of int 1 for both parameters. Furthermore, in the linear regression, an increase in the gene concentration with an increase of *int 1* was visible. This relationship between *int1* and virulence factors has also been found in *E*. *coli* isolates from river water [15]. The fact that the *int1* gene is ubiquitous in the pig farms studied could be associated with the spread of antibiotic resistance and virulence factors via horizontal gene transfer.

## Conclusions

The NMDS and LCA analyses used in this study yielded groups of farms with specific antibiotic use, and the corresponding patterns differed between clusters. Regression models revealed that amoxicillin, lincomycin, and enrofloxacin were the most probable cause of increased odds of the presence of prominent antibiotic resistance genes (*mcr1*, *dfrA1*, *bla*_*CTX-M*_) and bacterial virulence genes in many cases. However, direct effects of antibiotic use and corresponding resistance genes were uncommon. The association of integrase 1 with the *Escherichia*/*Shigella/Hafnia* group and *E*. *coli* fimbriae gene *fae* showed a possible link between multidrug resistance and virulence. The results confirm that multidrug-resistant enterobacteria are widespread in German pig farms, with varying patterns depending on age and farm. These findings give awareness of the impact of current antibiotic use while searching for alternative health strategies in modern pig production. More studies are necessary to figure out the effects of antibiotic use on virulence of bacteria.

## Supporting information

S1 Dataset Results (logarithmic copy number per gram feces of detected genes) of quantitative real-time PCR. (XLSX)

S1 File Supplementary S1-S8 Figs and S1-S39 Tables. (PDF)

## References

[1] Lekagul A, Tangcharoensathien V, Yeung S. Patterns of antibiotic use in global pig production: A systematic review. Veterinary and animal science. 2019;7:100058. Epub 2019/04/06. doi: 10.1016/j.vas.2019.100058 ; PubMed Central PMCID: PMC7386699. 32734079 PMC7386699

[2] Fairbrother JM, Nadeau E, Gyles CL. Escherichia coli in postweaning diarrhea in pigs: an update on bacterial types, pathogenesis, and prevention strategies. Anim Health Res Rev. 2005;6(1):17–39. doi: 10.1079/ahr2005105 . 16164007

[3] Lalles JP, Bosi P, Smidt H, Stokes CR. Weaning ‐ A challenge to gut physiologists. Livest Sci. 2007;108(1–3):82–93. doi: 10.1016/j.livsci.2007.01.091. PubMed PMID: WOS:000247123100020.

[4] World Health O. Critically important antimicrobials for human medicine. 6th rev. ed. Geneva: World Health Organization; 2019 2019.

[5] Liu YY, Wang Y, Walsh TR, Yi LX, Zhang R, Spencer J, et al. Emergence of plasmid-mediated colistin resistance mechanism MCR-1 in animals and human beings in China: a microbiological and molecular biological study. The Lancet Infectious diseases. 2016;16(2):161–8. Epub 2015/11/26. doi: 10.1016/S1473-3099(15)00424-7 . 26603172

[6] Irrgang A, Roschanski N, Tenhagen BA, Grobbel M, Skladnikiewicz-Ziemer T, Thomas K, et al. Prevalence of mcr-1 in E. coli from Livestock and Food in Germany, 2010–2015. PloS one. 2016;11(7):10. doi: 10.1371/journal.pone.0159863. PubMed PMID: WOS:000381515200055. 27454527 PMC4959773

[7] Perrin-Guyomard A, Bruneau M, Houée P, Deleurme K, Legrandois P, Poirier C, et al. Prevalence of mcr-1 in commensal Escherichia coli from French livestock, 2007 to 2014. Euro Surveill. 2016;21(6). Epub 2016/02/24. doi: 10.2807/1560-7917.ES.2016.21.6.30135 . 26898350

[8] Quesada A, Ugarte-Ruiz M, Iglesias MR, Porrero MC, Martínez R, Florez-Cuadrado D, et al. Detection of plasmid mediated colistin resistance (MCR-1) in Escherichia coli and Salmonella enterica isolated from poultry and swine in Spain. Res Vet Sci. 2016;105:134–5. Epub 2016/04/02. doi: 10.1016/j.rvsc.2016.02.003 . 27033921

[9] Rhouma M, Beaudry F, Letellier A. Resistance to colistin: what is the fate for this antibiotic in pig production? Int J Antimicrob Agents. 2016;48(2):119–26. Epub 2016/05/29. doi: 10.1016/j.ijantimicag.2016.04.008 . 27234675

[10] Geser N, Stephan R, Hächler H. Occurrence and characteristics of extended-spectrum β-lactamase (ESBL) producing Enterobacteriaceae in food producing animals, minced meat and raw milk. BMC Vet Res. 2012;8:21. Epub 2012/03/09. doi: 10.1186/1746-6148-8-21 ; PubMed Central PMCID: PMC3319423. 22397509 PMC3319423

[11] Ewers C, Bethe A, Semmler T, Guenther S, Wieler LH. Extended-spectrum β-lactamase-producing and AmpC-producing Escherichia coli from livestock and companion animals, and their putative impact on public health: a global perspective. Clinical microbiology and infection: the official publication of the European Society of Clinical Microbiology and Infectious Diseases. 2012;18(7):646–55. Epub 2012/04/24. doi: 10.1111/j.1469-0691.2012.03850.x . 22519858

[12] Michael GB, Kaspar H, Siqueira AK, de Freitas Costa E, Corbellini LG, Kadlec K, et al. Extended-spectrum β-lactamase (ESBL)-producing Escherichia coli isolates collected from diseased food-producing animals in the GERM-Vet monitoring program 2008–2014. Veterinary microbiology. 2017;200:142–50. Epub 2016/09/17. doi: 10.1016/j.vetmic.2016.08.023 . 27634182

[13] Gillings MR. Evolutionary consequences of antibiotic use for the resistome, mobilome and microbial pangenome. Front Microbiol. 2013;4:4. Epub 2013/02/07. doi: 10.3389/fmicb.2013.00004 ; PubMed Central PMCID: PMC3560386. 23386843 PMC3560386

[14] Gillings MR. Integrons: past, present, and future. Microbiol Mol Biol Rev. 2014;78(2):257–77. Epub 2014/05/23. doi: 10.1128/MMBR.00056-13 ; PubMed Central PMCID: PMC4054258. 24847022 PMC4054258

[15] Koczura R, Mokracka J, Barczak A, Krysiak N, Kaznowski A. Association between the presence of class 1 integrons, virulence genes, and phylogenetic groups of Escherichia coli isolates from river water. Microbial ecology. 2013;65(1):84–90. Epub 2012/08/21. doi: 10.1007/s00248-012-0101-3 ; PubMed Central PMCID: PMC3541932. 22903163 PMC3541932

[16] Guillard T, Pons S, Roux D, Pier GB, Skurnik D. Antibiotic resistance and virulence: Understanding the link and its consequences for prophylaxis and therapy. Bioessays. 2016;38(7):682–93. Epub 20160601. doi: 10.1002/bies.201500180 . 27248008

[17] Wallmann J, Bode C, Köper L, Heberer T. Abgabemengenerfassung von Antibiotika in Deutschland 2019. Deutsches Tierärzteblatt. 2020.

[18] Speksnijder DC, Wagenaar JA. Reducing antimicrobial use in farm animals: how to support behavioral change of veterinarians and farmers. Anim Front. 2018;8(2):4–9. Epub 2018/06/07. doi: 10.1093/af/vfy006 ; PubMed Central PMCID: PMC6951992. 32002213 PMC6951992

[19] Songer JG, Uzal FA. Clostridial enteric infections in pigs. Journal of veterinary diagnostic investigation: official publication of the American Association of Veterinary Laboratory Diagnosticians, Inc. 2005;17(6):528–36. Epub 2006/02/16. doi: 10.1177/104063870501700602 . 16475510

[20] Larsson J, Aspan A, Lindberg R, Grandon R, Baverud V, Fall N, et al. Pathological and bacteriological characterization of neonatal porcine diarrhoea of uncertain aetiology. J Med Microbiol. 2015;64:916–26. doi: 10.1099/jmm.0.000108. PubMed PMID: WOS:000362699500015. 26272503

[21] World Health O, Food, Agriculture Organization of the United N. Interventions for the control of non-typhoidal Salmonella spp. in beef and pork: meeting report and systematic review. Geneva: World Health Organization; 2016 2016.

[22] Barilli E, Bacci C, StellaVilla Z, Merialdi G, D’Incau M, Brindani F, et al. Antimicrobial resistance, biofilm synthesis and virulence genes in Salmonella isolated from pigs bred on intensive farms. Italian journal of food safety. 2018;7(2):7223. Epub 2018/07/27. doi: 10.4081/ijfs.2018.7223 ; PubMed Central PMCID: PMC6036996. 30046559 PMC6036996

[23] Lührmann A, Ovadenko K, Hellmich J, Sudendey C, Belik V, Zentek J, et al. Characterization of the fecal microbiota of sows and their offspring from German commercial pig farms. PloS one. 2021;16(8):e0256112. Epub 20210816. doi: 10.1371/journal.pone.0256112 ; PubMed Central PMCID: PMC8367078. 34398927 PMC8367078

[24] Perreten V, Boerlin P. A new sulfonamide resistance gene (sul3) in Escherichia coli is widespread in the pig population of Switzerland. Antimicrobial agents and chemotherapy. 2003;47(3):1169–72. doi: 10.1128/AAC.47.3.1169-1172.2003 ; PubMed Central PMCID: PMC149312. 12604565 PMC149312

[25] Zhang X, Li X, Wang W, Qi J, Wang D, Xu L, et al. Diverse Gene Cassette Arrays Prevail in Commensal Escherichia coli From Intensive Farming Swine in Four Provinces of China. Front Microbiol. 2020;11:565349. Epub 20201014. doi: 10.3389/fmicb.2020.565349 ; PubMed Central PMCID: PMC7591504. 33154738 PMC7591504

[26] de Los Santos E, Laviña M, Poey ME. Strict relationship between class 1 integrons and resistance to sulfamethoxazole in Escherichia coli. Microb Pathog. 2021;161(Pt A):105206. Epub 20211004. doi: 10.1016/j.micpath.2021.105206 . 34619311

[27] Kaper JB, Nataro JP, Mobley HL. Pathogenic Escherichia coli. Nature reviews Microbiology. 2004;2(2):123–40. doi: 10.1038/nrmicro818 . 15040260

[28] Keel MK, Songer JG. The comparative pathology of Clostridium difficile-associated disease. Veterinary pathology. 2006;43(3):225–40. Epub 2006/05/05. doi: 10.1354/vp.43-3-225 . 16672570

[29] Roschanski N, Falgenhauer L, Grobbel M, Guenther S, Kreienbrock L, Imirzalioglu C, et al. Retrospective survey of mcr-1 and mcr-2 in German pig-fattening farms, 2011–2012. Int J Antimicrob Agents. 2017;50(2):266–71. doi: 10.1016/j.ijantimicag.2017.03.007. PubMed PMID: WOS:000406495300025. 28545990

[30] Miguela-Villoldo P, Moreno MA, Rodríguez-Lázaro D, Gallardo A, Hernández M, Serrano T, et al. Longitudinal study of the mcr-1 gene prevalence in Spanish food-producing pigs from 1998 to 2021 and its relationship with the use of polymyxins. Porcine Health Manag. 2022;8(1):12. Epub 20220317. doi: 10.1186/s40813-022-00255-0 ; PubMed Central PMCID: PMC8932235. 35300732 PMC8932235

[31] Fu H, He M, Wu J, Zhou Y, Ke S, Chen Z, et al. Deep Investigating the Changes of Gut Microbiome and Its Correlation With the Shifts of Host Serum Metabolome Around Parturition in Sows. Front Microbiol. 2021;12:729039. Epub 20210917. doi: 10.3389/fmicb.2021.729039 ; PubMed Central PMCID: PMC8484970. 34603257 PMC8484970

[32] Schaekel F, May T, Seiler J, Hartmann M, Kreienbrock L. Antibiotic drug usage in pigs in Germany-Are the class profiles changing? PloS one. 2017;12(8):15. doi: 10.1371/journal.pone.0182661. PubMed PMID: WOS:000408370700009. 28841685 PMC5571922

[33] van Rennings L, von Munchhausen C, Ottilie H, Hartmann M, Merle R, Honscha W, et al. Cross-Sectional Study on Antibiotic Usage in Pigs in Germany. PloS one. 2015;10(3):28. doi: 10.1371/journal.pone.0119114. PubMed PMID: WOS:000352138500075. 25785688 PMC4364977

[34] Smits WK, Lyras D, Lacy DB, Wilcox MH, Kuijper EJ. Clostridium difficile infection. Nat Rev Dis Primers. 2016;2:16020. Epub 20160407. doi: 10.1038/nrdp.2016.20 ; PubMed Central PMCID: PMC5453186. 27158839 PMC5453186

[35] Hromada S, Qian Y, Jacobson TB, Clark RL, Watson L, Safdar N, et al. Negative interactions determine Clostridioides difficile growth in synthetic human gut communities. Mol Syst Biol. 2021;17(10):e10355. doi: 10.15252/msb.202110355 ; PubMed Central PMCID: PMC8543057. 34693621 PMC8543057

[36] Tang S, Zhang S, Zhong R, Su D, Xia B, Liu L, et al. Time-course alterations of gut microbiota and short-chain fatty acids after short-term lincomycin exposure in young swine. Appl Microbiol Biotechnol. 2021;105(21–22):8441–56. Epub 20211015. doi: 10.1007/s00253-021-11627-x . 34651253

[37] Pouwels KB, Freeman R, Muller-Pebody B, Rooney G, Henderson KL, Robotham JV, et al. Association between use of different antibiotics and trimethoprim resistance: going beyond the obvious crude association. The Journal of antimicrobial chemotherapy. 2018;73(6):1700–7. doi: 10.1093/jac/dky031 . 29394363

[38] Kecerova Z, Cizek A, Nyc O, Krutova M. Clostridium difficile isolates derived from Czech horses are resistant to enrofloxacin; cluster to clades 1 and 5 and ribotype 033 predominates. Anaerobe. 2019;56:17–21. Epub 2019/01/11. doi: 10.1016/j.anaerobe.2019.01.005 . 30630037

[39] Candela T, Marvaud JC, Nguyen TK, Lambert T. A cfr-like gene cfr(C) conferring linezolid resistance is common in Clostridium difficile. Int J Antimicrob Agents. 2017;50(3):496–500. Epub 2017/07/01. doi: 10.1016/j.ijantimicag.2017.03.013 . 28663118

[40] Grześkowiak Ł, Zentek J, Vahjen W. Determination of the extent of Clostridium difficile colonisation and toxin accumulation in sows and neonatal piglets. Anaerobe. 2016;40:5–9. Epub 2016/04/26. doi: 10.1016/j.anaerobe.2016.04.012 . 27108595

[41] Martínez JL. Antibiotics and antibiotic resistance genes in natural environments. Science (New York, NY). 2008;321(5887):365–7. Epub 2008/07/19. doi: 10.1126/science.1159483 . 18635792

[42] Zhang L, Levy K, Trueba G, Cevallos W, Trostle J, Foxman B, et al. Effects of selection pressure and genetic association on the relationship between antibiotic resistance and virulence in Escherichia coli. Antimicrobial agents and chemotherapy. 2015;59(11):6733–40. Epub 20150817. doi: 10.1128/AAC.01094-15 ; PubMed Central PMCID: PMC4604409. 26282415 PMC4604409

