# Supplementary material for: Antimicrobial resistance- and pathogen patterns in the fecal microbiota of sows and their offspring in German commercial pig farms
Source: PLoS One. 2023 Aug 24;18(8):e0290554. doi: 10.1371/journal.pone.0290554 (PMC10449214; doi:10.1371/journal.pone.0290554)
Supplement: S1 File — (PDF) [file pone.0290554.s002.pdf]

Supporting information

1 Figures

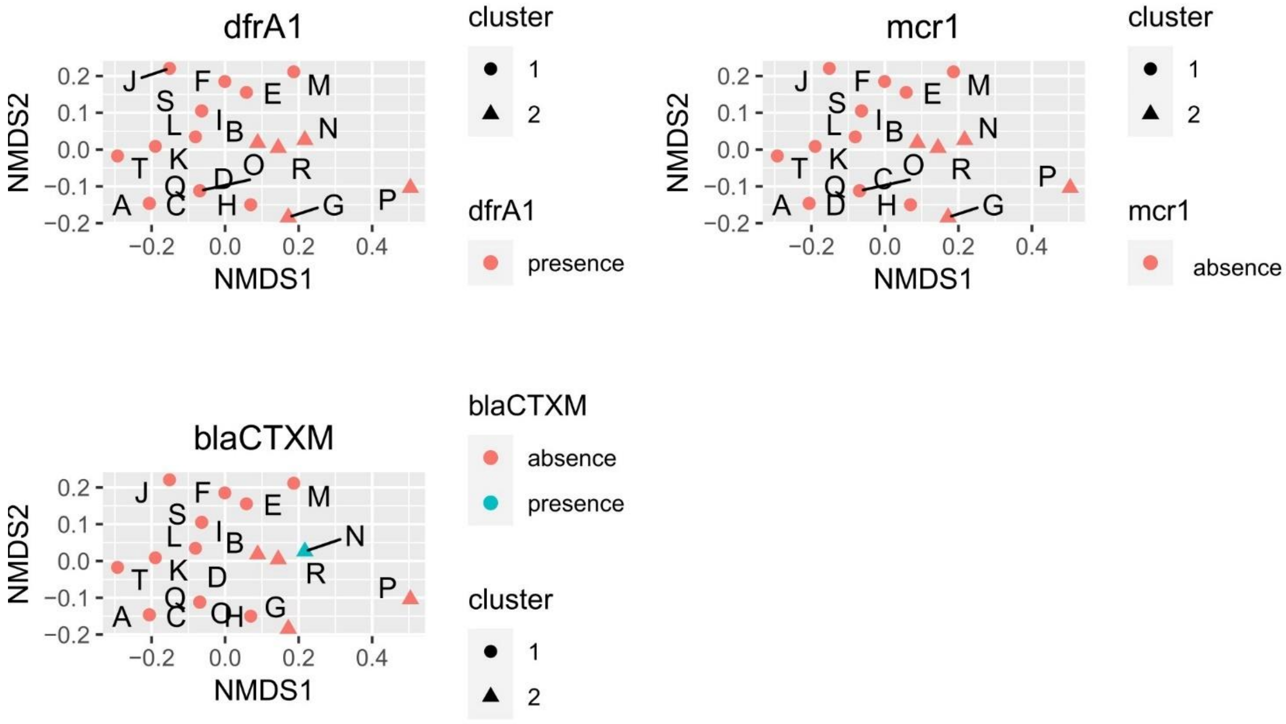

S1 Fig. Distribution of trimethoprim resistance (*dfrA1*), colistin resistance (*mcr-1*), and ESBL (*bla*$_{CTX\text{-}M}$) in clusters of antepartum sows clustered by antibiotic use.

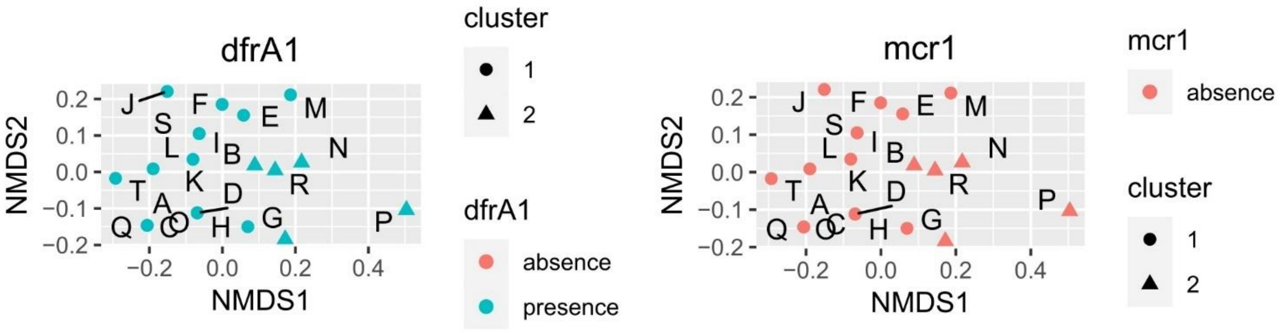

S2 Fig. Distribution of trimethoprim resistance (*dfrA1*) and colistin resistance (*mcr-1*) in clusters of postpartum sows clustered by antibiotic use.

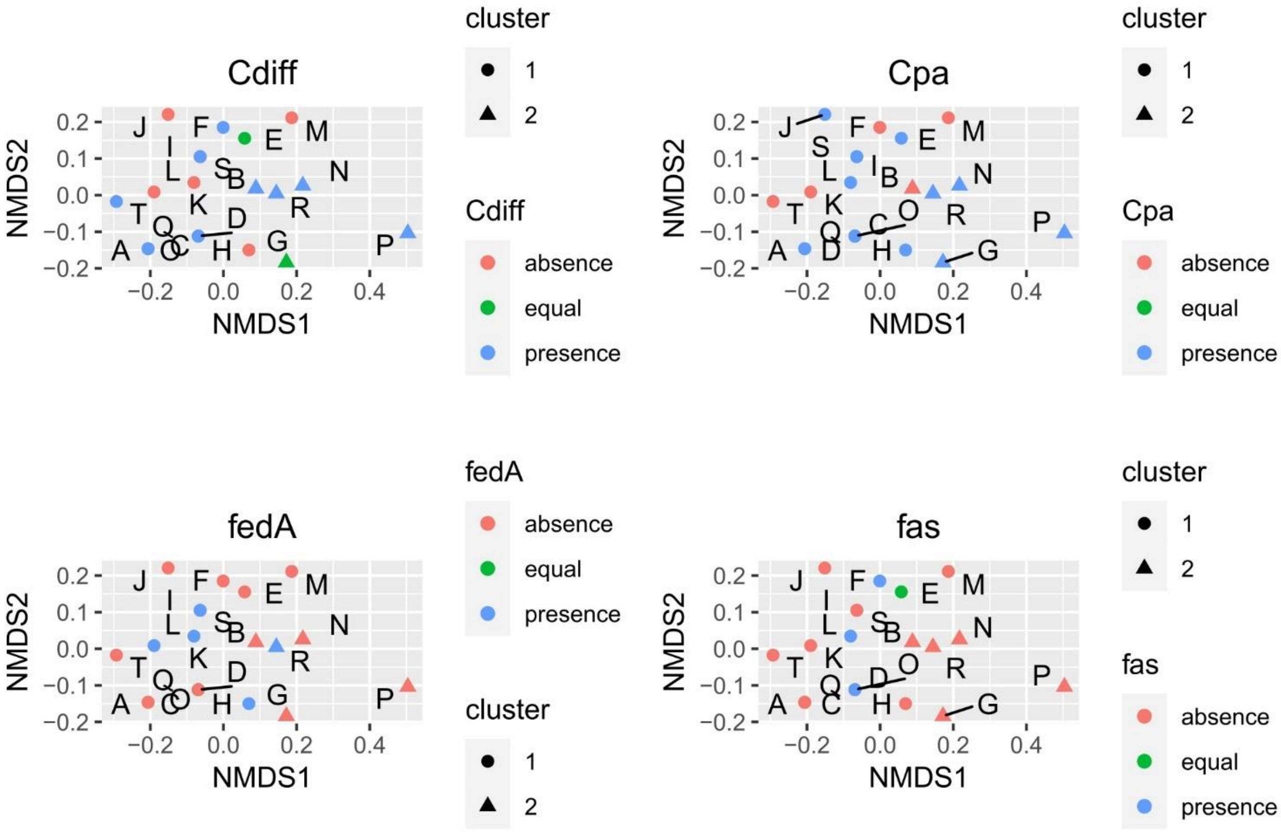

S3 Fig. Distribution of *C. difficile* (*Cdiff*), *C. perfringens* (*Cpa*), *E. coli* F18 (*fedA*) and *E. coli* F4 (*fas*) in clusters of antepartum sows clustered by antibiotic use.

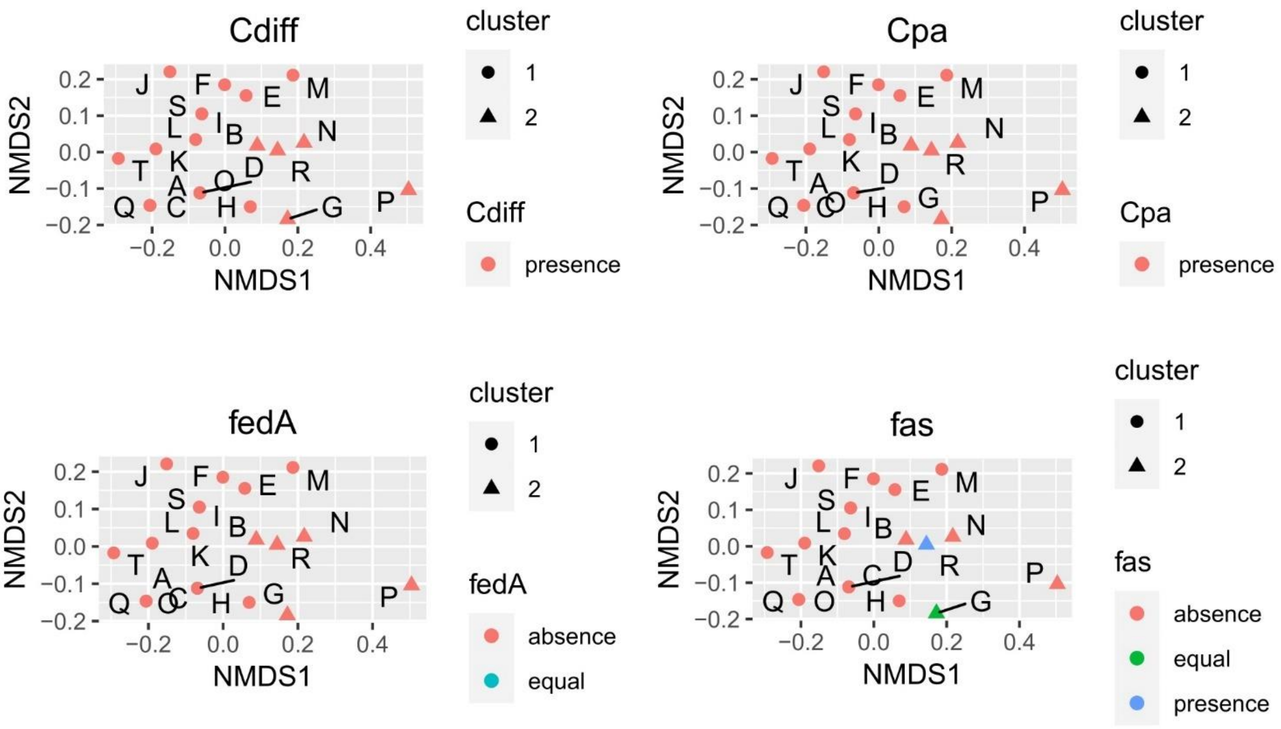

S4 Fig. Distribution of *C. difficile* (*Cdiff*), *C. perfringens* (*Cpa*), *E. coli* F18 (*fedA*) and *E. coli* F4 (*fas*) in clusters of antepartum sows clustered by antibiotic use.

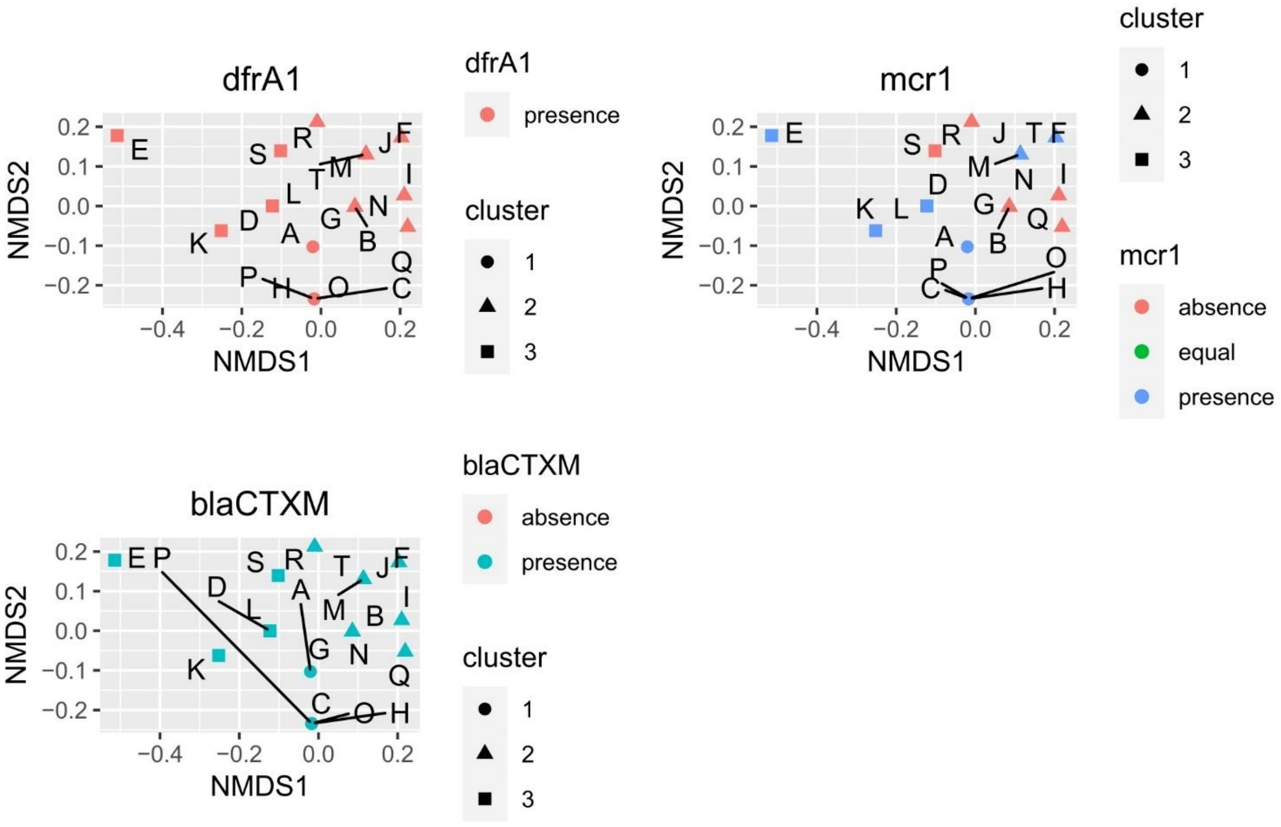

S5 Fig. Distribution of trimethoprim resistance (*dfrA1*), colistin resistance (*mcr-1*), and ESBL (*bla*$_{CTX-M}$) in clusters of suckling piglets clustered by antibiotic use.

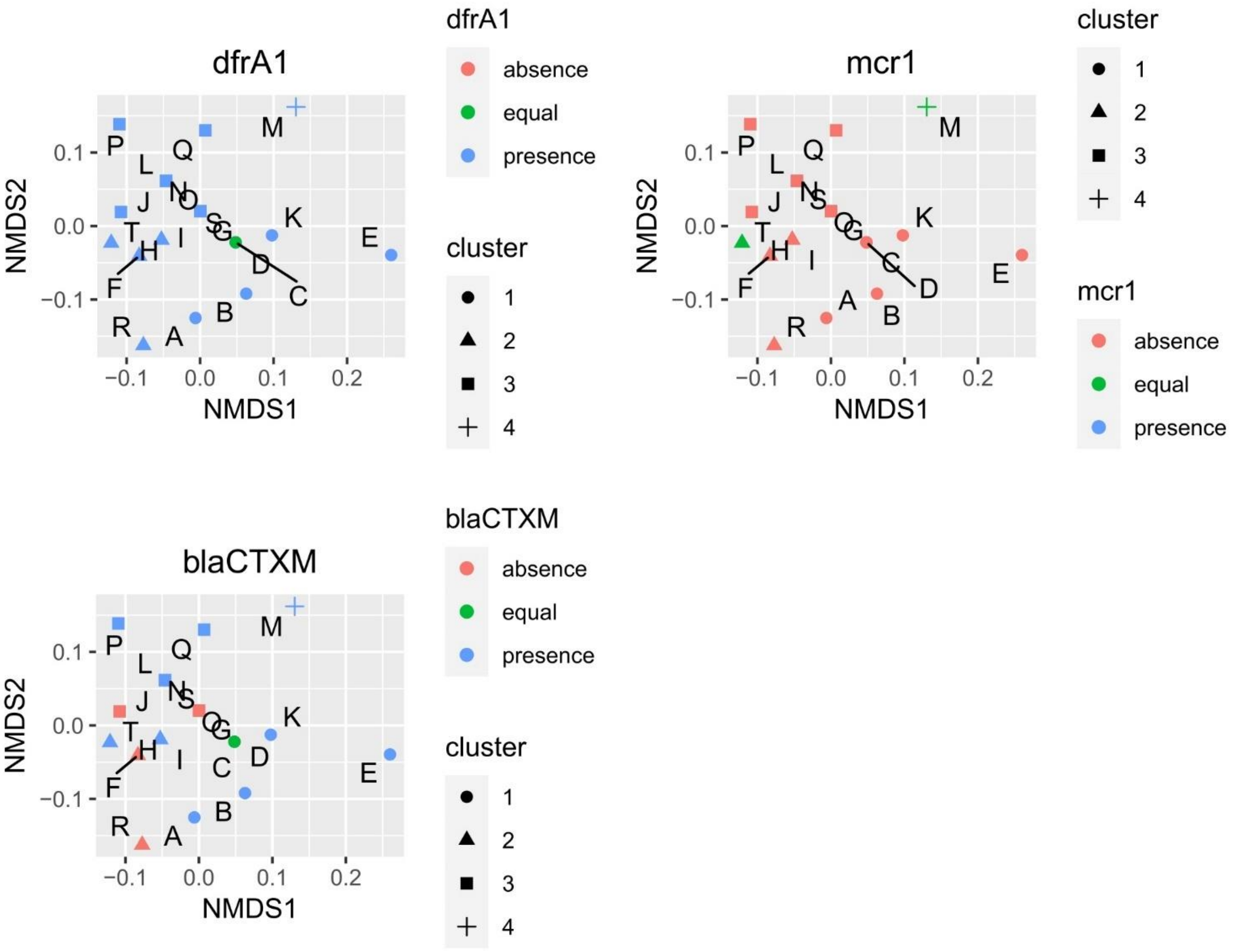

S6 Fig. Distribution of trimethoprim resistance (*dfrA1*), colistin resistance (*mcr-1*), and ESBL (*bla*$_{CTX\text{-}M}$) in clusters of weaned piglets clustered by antibiotic use.

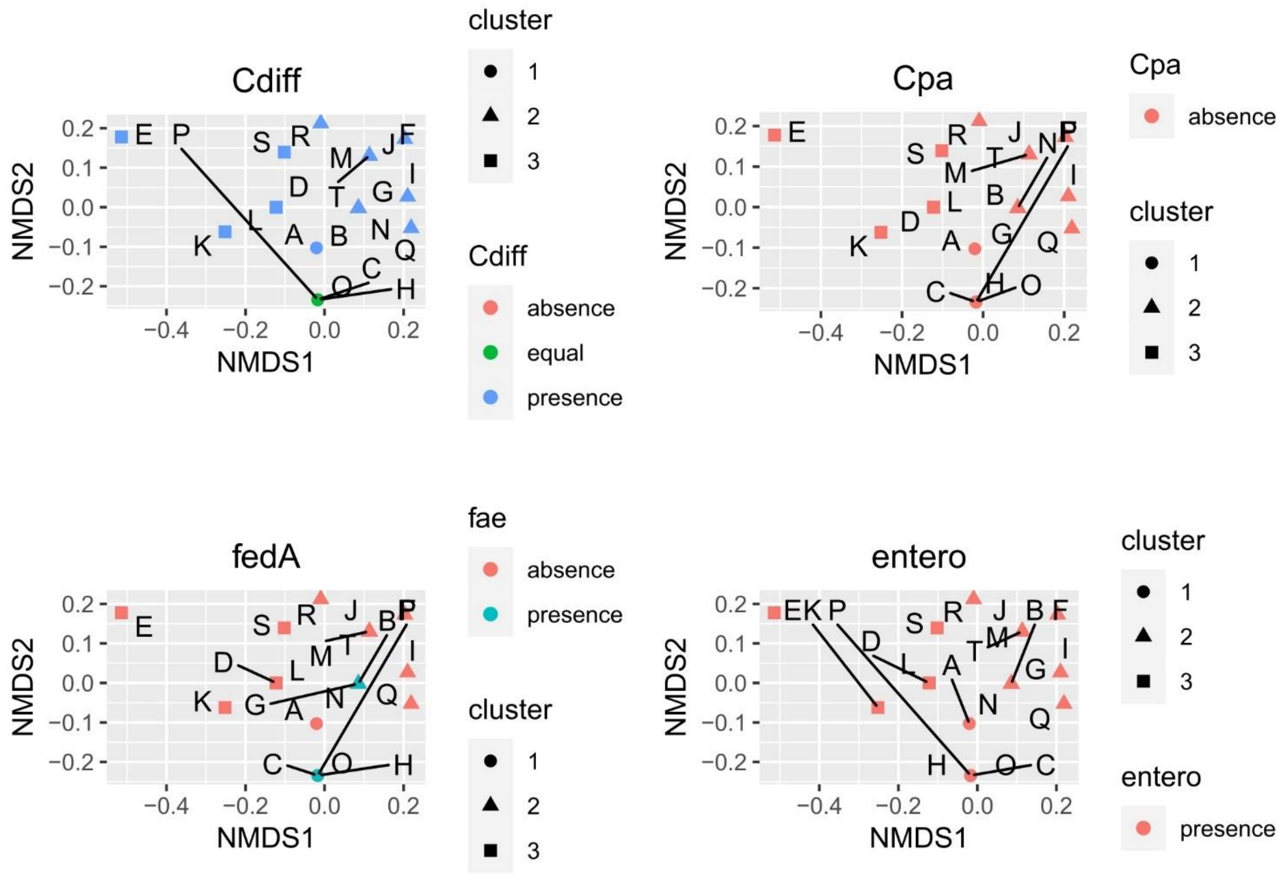

S7 Fig. Distribution of *C. difficile* (*Cdiff*), *C. perfringens* (*Cpa*), *E. coli* F18 (*fedA*), *Escherichia/Shigella/Hafnia* (*entero*) in clusters of suckling piglets clustered by antibiotic use.

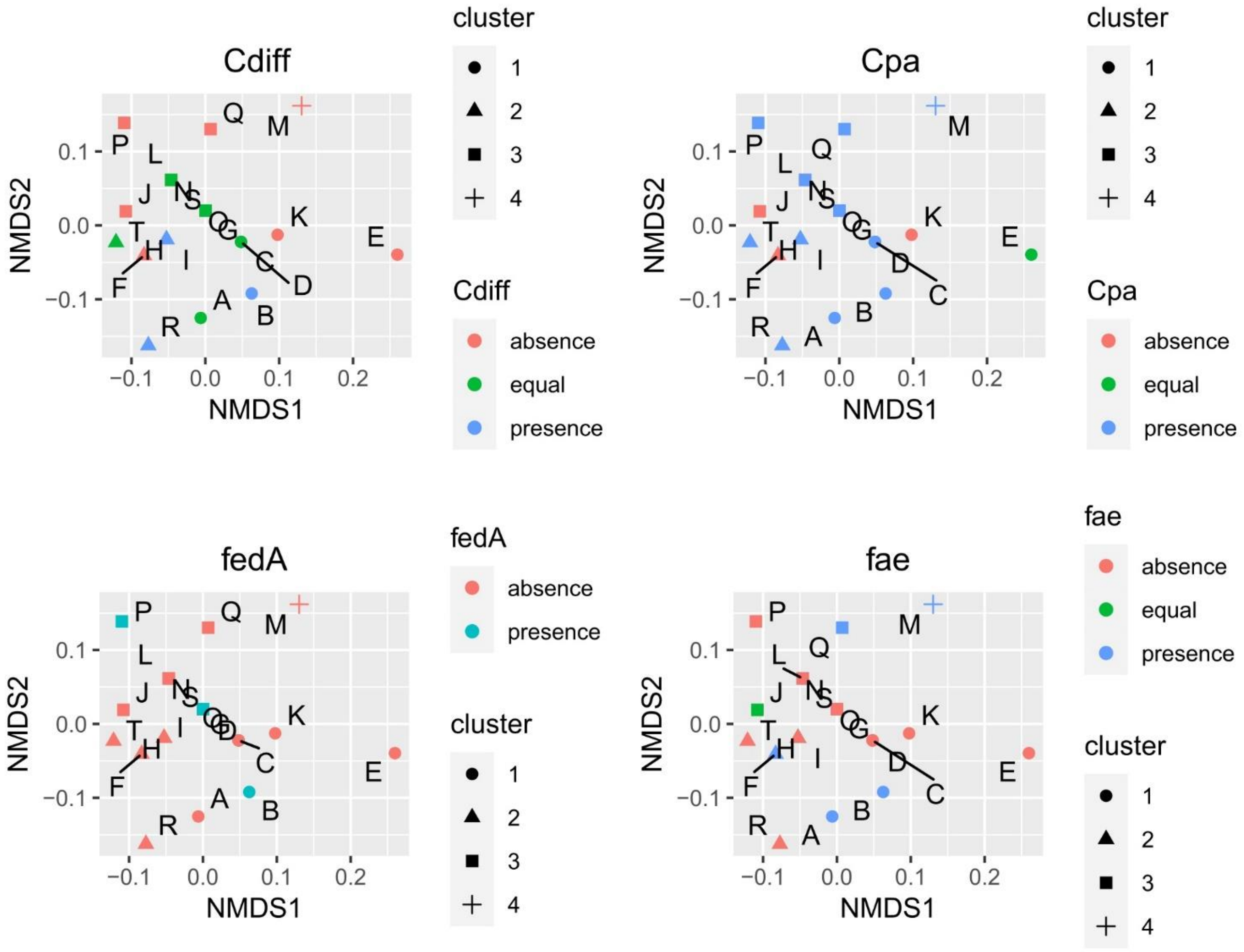

S8 Fig. Distribution of *C. difficile* (*Cdiff*), *C. perfringens* (*Cpa*), *E. coli* F18 (*fedA*), and *E. coli* F6 (*fae*) in clusters of weaned piglets clustered by antibiotic use.

2 Tables

S1 Table. Primer sequences, Annealing temperatures, and product sizes of target genes.

Target gene	Primer Name	Sequence	Annealing (°C)	Product size (bp)	Ref.
Colistin	mcr-1-f	5'-GGGCCTGCGTATTTTAAGCG-3'			
resistance	mcr-1-r	5'-CATAGGCATTGCTGTGCGTC-3'	50	186	(2)
ESBL	bla_{CTX-M}-f	5'-CGATGTGCAGTACCAGTAA-3'			
	bla_{CTX-M}-r	5'-TTAGTGACCAGAATCAGCGG-3'	50	591	(4)
Integrase 1	Int1-f	5'-AGGATGCGAACCACTTCATC-3'			
	Int1-r	5'-GCTGTTCTTCTACGGCAAGG-3'	56	840	(1)
Sulfonamide	Sul1-3-f	5'-CGATCCGGGGATGGGATTTT-3'			
resistance	Sul1-3-r	5'-CACCGAGACCAATAGCGGAA-3'	60	112	I*
Trimethoprim	dfrA1-f	5'-GGAGTGCCAAAGGTGAACAGC-3'			
resistance	dfrA1-r	5'-GAGGCGAAGTCTTGGGTAAAAAC-3'	50	356	(3)
C. difficile 16S	Cdiff-16S-1f	5'-TTGAGCGATTTACTTCGGTAAAGA-3'			
rRNA	Cdiff-16S-1r	5'-CCATCCTGTACTGGCTCACCT-3'	58	89	(7)
C. perfingens	cpa1-f	5'-TGACACAGGGGAATCACAAA-3'			
alpha toxin	cpa1-r	5'-CGCTATCAACGGCAGTAACA-3'	55	238	I*
E. coli F4	fas-f	5'-GTAACTCCACCGTTTGTATC-3'			
fimbriae	fas-r	5'-AAGTTACTGCCAGTCTATGC-3'	55	409	(8)
E. coli F18	fedA-f	5'-TGGTAACGTATCAGCAACTA-3'			
fimbriae	fedA-r	5'-ACTTACAGTGCTATTCGACG-3'	55	313	(8)
E. coli F4	Fae-f	5'-GTTGGTACAGGTCTTAATGG-3'			
fimbriae	Fae-r	5'-GAATCTGTCCGAGAATATCA-3'	55	499	(8)
16S rRNA	entero-f	5'-GTTAATACCTTTGCTCATTGA-3'			
Esherichia/	entero-r	5'ACCAGGGTATCTAATCCTGTT-3'			
Shigella/Hafnia			50	340	(5)
Salmonella	QVR-133	5'-GAAGCAGCGCCTGTAAAATC-3'			
enterotoxin	QVR-134	5'-TGGCTGTGGTGCAAAATATC-3'	60	198	(6)

*Institute of Animal Nutrition, Freie Universität, Berlin

1. Weldhagen GF. Stability of GES-type extended-spectrum ß-lactamase genes during antibiotic exposure. Southern African Journal of Epidemiology and Infection. 2015;22(4):119-22.
2. Hembach N, Schmid F, Alexander J, Hiller C, Rogall ET, Schwartz T. Occurrence of the mcr-1 Colistin Resistance Gene and other Clinically Relevant Antibiotic Resistance Genes in Microbial Populations at Different Municipal Wastewater Treatment Plants in Germany. Frontiers in Microbiology. 2017;8:11.
3. Toro CS, Farfan M, Contreras I, Flores O, Navarro N, Mora GC, et al. Genetic analysis of antibiotic-resistance determinants in multidrug-resistant Shigella strains isolated from Chilean children. Epidemiol Infect. 2005;133(1):81-6.
4. Roschanski N, Fischer J, Guerra B, Roesler U. Development of a multiplex real-time PCR for the rapid detection of the predominant beta-lactamase genes CTX-M, SHV, TEM and CIT-type AmpCs in Enterobacteriaceae. PloS one. 2014;9(7):e100956.
5. Malinen E, Kassinen A, Rinttilä T, Palva A. Comparison of real-time PCR with SYBR Green I or 5'-nuclease assays and dot-blot hybridization with rDNA-targeted oligonucleotide probes in quantification of selected faecal bacteria. Microbiology (Reading). 2003;149(Pt 1):269-77.
6. Riyaz-Ul-Hassan S, Verma V, Qazi GN. Real-time PCR-based rapid and culture-independent detection of Salmonella in dairy milk--addressing some core issues. Letters in applied microbiology. 2013;56(4):275-82.
7. Penders J, Vink C, Driessen C, London N, Thijs C, Stobberingh EE. Quantification of Bifidobacterium spp., Escherichia coli and Clostridium difficile in faecal samples of breast-fed and formula-fed infants by real-time PCR. FEMS Microbiol Lett. 2005;243(1):141-7.
8. Casey TA, Bosworth BT. Design and evaluation of a multiplex polymerase chain reaction assay for the simultaneous identification of genes for nine different virulence factors associated with Escherichia coli that cause diarrhea and edema disease in swine. Journal of veterinary diagnostic investigation : official publication of the American Association of Veterinary Laboratory Diagnosticians, Inc. 2009;21(1):25-30.

S2 Table. Overall concentration of antibiotic resistance genes, enterobacterial genes, and virulence genes in sow and piglet fecal samples of 20 pig farms [log copy number/g sample] (n=802).

**Farm**	***Salmonella***	***C. perfringens***	***C. difficile***	***Escherichia***	***fedA***	***fae***	***fas***	***dfrA1***	***sul1-3***	***bla_{CTX-M}***	***mcr-1***	***int1***
**A**	n.d.	6.39	5.22	8.68	4.47	6.61	4.53	6.23	7.97	6.87	4.11	8.09
**B**	n.d.	5.46	5.46	8.89	5.27	6.34	4.25	6.44	7.74	5.72	4.53	7.64
**C**	4.20	6.89	5.13	8.73	5.06	5.70	3.72	5.48	7.54	5.20	3.80	7.50
**D**	3.59	5.92	4.78	8.61	5.34	n.d.	4.64	5.77	7.74	5.35	3.64	7.35
**E**	4.17	6.89	4.78	8.21	3.73	4.19	4.54	6.58	7.84	5.48	3.85	7.77
**F**	4.20	5.44	4.78	8.25	8.08	6.00	4.83	6.20	7.93	5.80	4.13	7.88
**G**	3.92	6.67	4.98	8.39	3.20	n.d.	4.23	5.19	7.48	5.35	3.50	7.60
**H**	4.52	6.41	4.86	8.05	4.67	7.94	4.09	6.09	8.14	5.16	2.21	8.23
**I**	3.66	6.75	5.14	8.36	3.88	6.38	4.05	5.83	7.56	5.88	3.85	7.60
**J**	4.58	5.97	3.96	8.41	4.63	6.62	4.30	5.72	7.46	4.88	3.62	7.64
**K**	4.29	6.32	4.90	8.34	4.27	4.07	4.10	6.15	8.13	5.93	3.39	8.11
**L**	3.83	6.50	4.22	7.45	4.64	5.40	4.04	6.09	7.60	5.41	2.94	7.66
**M**	3.48	6.37	4.71	8.23	3.46	6.33	3.83	6.02	7.79	6.19	3.25	7.85
**N**	4.60	6.67	4.35	8.37	4.38	5.83	4.03	5.87	7.74	5.12	3.12	7.55
**O**	n.d.	6.41	4.15	8.24	4.46	3.99	4.04	5.65	7.76	5.20	2.70	7.63
**P**	3.19	5.92	5.22	7.80	3.98	6.74	3.92	6.02	7.98	5.10	2.98	7.70
**Q**	3.27	5.78	5.29	8.05	5.94	6.40	4.20	5.67	7.53	5.11	3.45	7.98
**R**	4.20	6.76	4.73	8.26	6.02	5.13	4.84	5.30	7.80	4.63	3.92	7.62
**S**	n.d.	5.73	4.86	8.77	4.18	6.98	4.83	6.05	7.78	4.92	3.28	7.90
**T**	n.d.	5.67	5.03	8.72	2.87	5.84	5.41	7.05	8.24	4.99	4.19	8.24
**p- value***	0.524	0.001	0.000	0.000	0.007	0.022	0.022	0.000	0.000	0.000	0.018	0.000
**Total**	4.08	6.27	4.85	8.34	4.85	6.17	4.31	6.00	7.79	5.47	3.54	7.78
**SEM**	0.13	0.07	0.06	0.05	0.15	0.13	0.06	0.04	0.03	0.06	0.10	0.03

n.d. = not detected

* Kruskal- Wallis Test, ($p \leq 0.05$)

Salmonella = QVR gene, *Clostridium perfringens* = cpa gene, *Escherichia* = 16S rDNA of the phylogenetic *Escherichia/Hafnia/Shigella* group, *fedA* = *E. coli* fimbriae gene (F18), *fae* = *E. coli* fimbriae gene (F4), *fas* = *E. coli* fimbriae F6 gene; *sul1-3* = enterobacterial sulfonamide resistance gene, bla_{CTX-M} = enterobacterial ESBL gene, *mcr-1* = enterobacterial colistin gene, *int1* = enterobacterial integrase I gene

S3 Table. Overall prevalence of antibiotic resistance genes, enterobacterial genes, and virulence genes sow and piglet fecal samples of 20 pig farms [%] (n=802).

Farm	*bla_{CTX-M}*	*dfrA1*	*int1*	*mcr-1*	*sul1-3*	*C. difficile*	*C. perfringens*	*Escherichia*	*fae*	*fas*	*fedA*	*Salmonella*
**A**	66.67	94.87	100.00	28.21	100.00	58.97	69.23	100.00	25.64	12.82	10.26	0.00
**B**	40.00	85.00	100.00	40.00	100.00	72.50	90.00	100.00	30.00	10.00	25.00	0.00
**C**	35.90	46.15	100.00	17.95	100.00	82.05	89.74	89.74	10.26	17.95	28.21	7.69
**D**	42.50	75.00	100.00	17.50	100.00	82.50	82.50	87.50	0.00	17.50	42.50	12.50
**E**	60.00	92.50	100.00	27.50	100.00	62.50	80.00	100.00	2.50	30.00	5.00	2.50
**F**	34.15	92.68	100.00	21.95	100.00	63.41	60.98	100.00	41.46	21.95	7.32	4.88
**G**	45.00	75.00	100.00	12.50	95.00	67.50	90.00	100.00	0.00	10.00	12.50	15.00
**H**	40.00	92.50	100.00	15.00	95.00	70.00	95.00	97.50	12.50	20.00	25.00	12.50
**I**	60.00	85.00	100.00	10.00	100.00	70.00	95.00	95.00	15.00	15.00	25.00	5.00
**J**	27.50	90.00	100.00	30.00	100.00	60.00	80.00	100.00	15.00	30.00	12.50	12.50
**K**	36.59	90.24	100.00	31.71	90.24	63.41	85.37	100.00	2.44	31.71	26.83	14.63
**L**	45.00	85.00	100.00	47.50	97.50	62.50	62.50	97.50	17.50	12.50	22.50	2.50
**M**	77.50	85.00	100.00	35.00	100.00	57.50	77.50	100.00	20.00	12.50	10.00	2.50
**N**	85.71	83.33	100.00	16.67	100.00	66.67	92.86	100.00	40.48	9.52	11.90	7.14
**O**	46.34	82.93	100.00	21.95	97.56	46.34	97.56	95.12	2.44	31.71	19.51	0.00
**P**	60.00	92.50	100.00	35.00	97.50	72.50	92.50	100.00	42.50	25.00	12.50	2.50
**Q**	47.37	89.47	100.00	28.95	100.00	68.42	84.21	100.00	34.21	15.79	26.32	7.89
**R**	31.71	87.80	100.00	12.20	100.00	73.17	90.24	97.56	29.27	29.27	43.90	2.44
**S**	45.00	95.00	100.00	27.50	100.00	77.50	85.00	100.00	10.00	22.50	22.50	0.00
**T**	52.50	97.50	100.00	37.50	100.00	72.50	70.00	100.00	7.50	5.00	2.50	0.00
**Total**	49.00	85.91	100.00	25.69	98.63	67.46	83.54	98.00	17.96	19.08	19.58	5.61
**SEM**	1.77	1.23	0.00	1.54	0.41	1.66	1.31	0.49	1.36	1.39	1.40	0.81

Salmonella = *QVR* gene, *Clostridium perfringens* = *cpa* gene, *Escherichia* = 16S rDNA of the phylogenetic *Escherichia/Hafnia/Shigella* group, *fedA* = *E. coli* fimbriae gene (F18), *fae* = *E. coli* fimbriae gene (F4), *fas* = *E. coli* fimbriae F6 gene; *sul1-3* = enterobacterial sulfonamide resistance gene, *bla_{CTX-M}* = enterobacterial ESBL gene, *mcr-1* = enterobacterial colistin gene, *int1* = enterobacterial integrase I gene

S4 Table. Comparison between ante- and postpartum (AP, PP) sows for the concentration and prevalence of antibiotic resistance genes, enterobacterial genes, and virulence genes in sow samples of 20 pig farms ([log copy number/g sample, [%]).

	Concentration			Prevalence		
Target gene	AP	PP	p-value*	AP	PP	p-value**
blaCTXM	4.60	5.00	0.159	14.49[d]	38.69[c]	<0.001
dfrA1	5.63[b]	5.89[a]	0.019	92.27[c]	76.88[d]	<0.001
int1	7.63[a]	7.39[b]	<0.001	100.00	100.00	n.a
mcr-1	3.78[b]	4.90[a]	<0.001	14.01[c]	7.54[d]	0.036
sul1-3	7.62[b]	7.85[a]	0.001	94.69[d]	100.00[c]	0.001
C. difficile	4.43	4.47	0.111	58.94[d]	93.97[c]	<0.001
C. perfringens	5.68	5.83	0.402	66.67[d]	98.99[c]	<0.001
Escherichia	7.95[b]	8.52[a]	<0.001	100.00	100.00	n.a
fae	5.59	6.18	0.079	8.21	12.56	0.15
fas	4.58	4.46	0.183	23.19	19.10	0.313
fedA	3.81 [b]	4.49 [a]	<0.001	33.33 [c]	13.57 [d]	<0.001
Salmonella	4.26	3.78	0.370	7.73	5.03	0.266

*Mann-Whitney Test: a,b denotes significant difference for time point, ($p \leq 0.05$)
**Chi- squared Test: c,d denotes significant difference for time point, ($p \leq 0.05$)
n.a.= not available

Salmonella = *QVR* gene, *Clostridium perfringens* = *cpa* gene, *Escherichia* = 16S rDNA of the phylogenetic *Escherichia/Hafnia/Shigella* group, *fedA* = *E. coli* fimbriae gene (F18), *fae* = *E. coli* fimbriae gene (F4), *fas* = *E. coli* fimbriae F6 gene; *sul1-3* = enterobacterial sulfonamide resistance gene, $bla_{CTX\text{-}M}$ = enterobacterial ESBL gene, *mcr-1* = enterobacterial colistin gene, *int1* = enterobacterial integrase I gene

S5 Table. Comparison of suckling- and weaned piglets for the concentration and prevalence of antibiotic resistance genes, enterobacterial genes, and virulence genes in piglet samples of 20 pig farms ([log copy number/g sample, [%]).

	Concentration			Prevalence		
Target gene	Suckling	Weaned	p-value*	Suckling	Weaned	p-value**
blaCTXM	5.78[a]	5.55[b]	0.027	86.29[c]	58.29[d]	<0.001
dfrA1	6.16	6.35	0.154	90.36	83.92	0.056
int1	8.72[a]	7.38[b]	<0.001	100.00	100.00	n.a.
mcr-1	3.76[a]	2.69[b]	<0.001	52.28[c]	29.65[d]	<0.001
sul1-3	8.46[a]	7.22[b]	<0.001	100.00	100.00	n.a.
C. difficile	5.59[a]	5.03[b]	0.014	72.59[c]	44.72[d]	<0.001
C. perfringens	7.98[a]	5.05[b]	<0.001	99.49[c]	69.85[d]	<0.001
Escherichia	8.37[b]	8.52[a]	0.026	91.88[d]	100.00[c]	<0.001
fae	6.27	6.27	1.000	12.18 [d]	39.20 [c]	<0.001
fas	3.95	4.20	0.114	21.32[c]	12.56[d]	0.020
fedA	4.37 [b]	6.73 [a]	<0.001	7.11 [d]	23.62 [c]	<0.001
Salmonella	4.14	4.03	0.457	4.06	5.53	0.495

*Mann-Whitney Test: a,b denotes significant difference for time point, ($p \leq 0.05$)
**Chi- squared Test: c,d denotes significant difference for time point, ($p \leq 0.05$)
n.a.= not available

Salmonella = *QVR* gene, *Clostridium perfringens* = *cpa* gene, *Escherichia* = 16S rDNA of the phylogenetic *Escherichia/Hafnia/Shigella* group, *fedA* = *E. coli* fimbriae gene (F18), *fae* = *E. coli* fimbriae gene (F4), *fas* = *E. coli* fimbriae F6 gene; *sul1-3* = enterobacterial sulfonamide resistance gene, $bla_{CTX\text{-}M}$ = enterobacterial ESBL gene, *mcr-1* = enterobacterial colistin gene, *int1* = enterobacterial integrase I gene

S6 Table. **Results of the logistic regression model for the presence of *dfrA1* gene as the dependent variable in antepartum sows.**

Variable	Estimate	Std.Error	odds ratios	OR lower CI	OR upper CI	p-value
Intercept	2.49	0.43	12.08	5.20	28.05	<0.001
Amoxicillin (oral)	-2.76	1.07	0.06	0.01	0.51	0.001
Amoxicillin	2.00	0.82	7.42	1.47	37.38	0.015
Neomycin (oral)	-2.30	1.36	0.10	0.01	1.45	0.092
Trimethoprim	-1.25	0.64	0.29	0.08	1.00	0.050

*in combination with sulfadimidine

S7 Table. Results of the linear regression model for the concentration of *drfA1* gene as the dependent variable in antepartum sows.

Variable	Estimate	Std.Error	p-value
Intercept	5.4701	0.0937	>0.001
Amoxicillin	0.2113	0.1215	0.0835
Lincomycin	-0.3558	0.1701	0.0378
Trimethoprim*	0.2347	0.1366	0.0875
Tylosin	0.4508	0.2579	0.0821

*in combination with sulfadimidine

S8 Table. Results of the logistic regression model for the presence of *dfrA1* gene as the dependent variable in postpartum sows.

Variable	Estimate	Std.Error	odds ratios	OR lower CI	OR upper CI	p-value
Intercept	0.45	0.35	1.56	0.79	3.11	0.2013
Amoxicillin (oral)	1.93	0.83	6.87	1.36	34.65	0.0196
Amoxicillin	-0.64	0.46	0.53	0.22	1.29	0.1610
Enrofloxacin	1.03	0.51	2.81	1.03	7.62	0.0428
Florfenicol	1.49	0.59	4.43	1.39	14.13	0.0120
Trimethoprim*	1.11	0.53	3.03	1.08	8.53	0.0353
Tylosin	1.58	0.96	4.85	0.74	31.60	0.0989

*in combination with sulfadimidine

S9 Table. Results of the linear regression model for the concentration of *drfA1* gene as the dependent variable in postpartum sows.

Variable	Estimate	Std.Error	p-value
Intercept	5.19	0.14	<0.001
Amoxicillin (oral)	1.04	0.24	<0.001
Enrofloxacin	0.50	0.18	0.0058
Florfenicol	1.01	0.18	<0.001
Neomycin (oral)	0.78	0.31	0.0129
Trimethoprim*	0.51	0.16	0.0011

*in combination with sulfadimidine

S10 Table. Results of the logistic regression model for the presence of *bla*$_{CTX\text{-}M}$ gene as the dependent variable in postpartum sows.

Variable	Estimate	Std.Error	odds ratios	OR lower CI	OR upper CI	p-value
Intercept	-1.01	0.25	0.36	0.22	0.59	<0.001
Enrofloxacin	-1.83	0.76	0.16	0.04	0.71	0.0163
Florfenicol	1.37	0.41	3.95	1.77	8.84	<0.001
Lincomycin	4.34	0.86	76.67	14.22	413.43	<0.001
Neomycin (oral)	2.84	0.97	17.14	14.22	114.42	0.0033

S11 Table. Results of the linear regression model for the concentration of *bla*$_{CTX\text{-}M}$ gene as the dependent variable in postpartum sows.

Variable	Estimate	Std.Error	p-value
Intercept	5.44	0.20	<0.001
Amoxicillin (oral)	-0.81	0.46	0.0819
Enrofloxacin	-1.37	0.39	<0.001
Lincomycin	0.86	0.40	0.0353
Trimethoprim*	-0.85	0.32	0.0096

*in combination with sulfadimidine

S12 Table. **Results of the linear regression model for the concentration of *dfrA1* gene as the dependent variable in suckling piglets.**

Variable	Estimate	Std.Error	p-value
Intercept	5.2643	0.233	<0.001
Amoxicillin	0.983	0.2826	<0.001
Cefquinome	2.3757	0.5437	<0.001
Florfenicol	1.0659	0.5301	0.0459
Benzylpenicillin*	0.9941	0.3437	0.0043

*in combination with streptomycin

S13 Table. Results of the logistic regression model for the presence of *mcr-1* gene as the dependent variable in suckling piglets.

Variable	Estimate	Std.Error	odds ratios	OR lower CI	OR upper CI	p-value
Intercept	-0.15	0.32	0.86	0.46	1.61	0.6313
Amoxicillin	2.22	1.08	9.20	1.12	75.71	0.0390
Cefquinome	2.42	1.25	11.25	0.97	130.21	0.0527
Ceftiofur	-2.04	1.10	0.13	0.01	1.10	0.0637
Florfenicol	2.06	1.12	7.87	0.88	70.14	0.0644
Benzylpenicillin*	2.33	1.12	10.29	1.15	91.61	0.0367

*in combination with streptomycin

S14 Table. **Results of the linear regression model for the concentration of *mcr-1* gene as the dependent variable in suckling piglets.**

Variable	Estimate	Std.Error	p-value
Intercept	0.97	2.24	0.6661
Amoxicillin	0.50	0.22	0.0267
Cefquinome	0.74	0.46	0.1110
Ceftiofur	0.32	0.23	0.1690
Colistin	-0.85	0.42	0.0459

S15 Table. Results of the logistic regression model for the presence of *dfrA1* gene as the dependent variable in weaned piglets.

Variable	Estimate	Std.Error	odds ratios	OR lower CI	OR upper CI	p-value
Intercept	0.85	0.29	2.35	1.32	4.18	0.0037
Amoxicillin	1.06	0.42	2.89	1.26	6.61	0.0118
Amoxicillin (oral)	0.49	0.48	1.63	0.64	4.15	0.3062
Cefquinome	1.12	0.81	3.07	0.63	15.06	0.1660

S16 Table. Results of the beta linear regression model for the concentration of *dfrA1* gene as the dependent variable in weaned piglets.

Variable	Estimate	Std.Error	p-value
Intercept	1.22	1.26	0.3375
Amoxicillin (oral)	0.39	0.17	0.0198
Colistin (oral)	1.06	0.28	<0.001
Lincomycin	0.80	0.33	0.0166
Neomycin (oral)	0.64	0.31	0.0398

Table 17. Results of the logistic regression model for the presence of *mcr-1* gene as the dependent variable in weaned piglets.

Variable	Estimate	Std.Error	odds ratios	OR lower CI	OR upper CI	p-value
Intercept	-1.10	0.25	0.33	0.20	0.55	<0.001
Amoxicillin (oral)	-1.58	0.46	0.21	0.08	0.51	<0.001
Cefquinome	0.92	0.42	2.50	0.79	7.91	0.1189
Doxycycline (oral)	1.21	0.54	3.35	1.16	9.67	0.0251
Enrofloxacin	1.60	0.47	4.97	1.97	12.57	<0.001
Lincomycin	2.68	0.80	14.64	3.03	70.78	<0.001
Neomycin (oral)	-1.35	0.80	0.26	0.05	1.25	0.0928

S18 Table. Results of the beta linear regression model for the concentration of *mcr-1* gene as the dependent variable in weaned piglets.

Variable	Estimate	Std.Error	p-value
Intercept	0.58	0.22	0.0111
Amoxicillin (oral)	-0.97	0.49	0.0517
Cefquinome	0.89	0.42	0.0376
Doxycycline (oral)	1.71	0.63	0.0099
Lincomycin (oral)	-1.80	0.74	0.0195
Lincomycin	1.39	0.61	0.0286
Neomycin (oral)	-0.88	0.56	0.1254
Oxytetracycline	1.73	0.57	0.0041

S19 Table. Results of the logistic regression model for the presence of *bla_{CTX-M}* gene asthe dependent variable in weaned piglets.

Variable	Estimate	Std.Error	odds ratios	OR lower CI	OR upper CI	p-value
Intercept	0.19	0.17	1.21	0.86	1.68	0.2708
Cefquinome	1.20	0.58	3.32	1.05	10.42	0.0402
Doxycycline (oral)	-1.03	0.52	0.36	0.13	0.98	0.0452
Lincomycin (oral)	3.04	1.16	21.00	2.16	204.59	0.0087
Oxytetracycline	2.01	1.07	7.46	0.92	60.48	0.0598

S20 Table. Results of the beta linear regression model for the concentration of *bla_{CTX-M}* gene as the dependent variable in weaned piglets.

Variable	Estimate	Std.Error	p-value
Intercept	-2.33	0.69	0.0010
Amoxicillin	1.84	0.64	0.0052
Amoxicillin (oral)	0.73	0.33	0.0267
Cefquinome	3.25	0.73	<0.001
Colistin (oral)	3.85	0.85	<0.001
Lincomycin (oral)	-2.79	0.89	0.0023
Lincomycin	-1.01	0.57	0.0778
Oxytetracycline	2.78	0.81	<0.001

S21 Table. Results of the logistic regression model for the presence of *C. difficile* (16s-rDNA gene) as the dependent variable in antepartum sows.

Variable	Estimate	Std.Error	odds ratios	OR lower CI	OR upper CI	p-value
Intercept	0.9126	0.2392	2.4907	1.5584	3.9807	0.0001
Amoxicillin (oral)	-0.957	0.5192	0.384	0.1388	1.0624	0.0652
Florfenicol	-0.6583	0.3709	0.5177	0.2502	1.0711	0.0759
Lincomycin	-0.7506	0.436	0.472	0.2008	1.1096	0.0851
Trimethoprim	-0.6695	0.3376	0.5119	0.2641	0.9922	0.0473

*in combination with sulfadimidine

S22 Table. Results of the beta linear regression model for the concentration of *C. difficile* (16s-rDNA gene) as the dependent variable in antepartum sows.

Variable	Estimate	Std.Error	p-value
Intercept	0.3005	0.0587	<0.001
Amoxicillin (oral)	0.2555	0.1584	0.1102
Enrofloxacin	0.2875	0.0923	0.0024
Lincomycin	-0.5267	0.1207	<0.001

S23 Table. Results of the logistic regression model for the presence of *E. coli* F18 fimbriae (*fedA*) as the dependent variable in antepartum sows.

Variable	Estimate	Std.Error	odds ratios	OR lower CI	OR upper CI	p-value
Intercept	-0.7053	0.1909	0.4939	0.3397	0.7181	0.0002
Amoxicillin (oral)	-1.7245	1.0604	0.1782	0.0223	1.4246	0.1039
Enrofloxacin	1.1908	0.413	3.2897	1.4642	7.3912	0.0039
Lincomycin	-1.5105	0.5926	0.2207	0.0691	0.7054	0.0108
Neomycin (oral)	-1.8718	0.8713	0.1538	0.0278	0.8486	0.0316

S24 Table. Results of the beta linear regression model for the concentration of *E. coli* F18 fimbriae (*fedA*) as the dependent variable in antepartum sows.

Variable	Estimate	Std.Error	p-value
Intercept	4.5803	2.5462	0.0776
Amoxicillin (oral)	-0.6014	0.3242	0.0690
Amoxicillin	-1.3458	0.8617	0.1241
Enrofloxacin	-1.1939	0.5013	0.0208
Florfenicol	-1.2416	0.5658	0.0325
Lincomycin	-1.4971	0.5485	0.0085
Trimethoprim*	-1.3322	0.5115	0.0118

*in combination with sulfadimidine

S25 Table. Results of the logistic regression model for the presence of *E. coli* F4 fimbriae (*fas*) as the dependent variable in antepartum sows.

Variable	Estimate	Std.Error	odds ratios	OR lower CI	OR upper CI	p-value
Intercept	-1.1058	0.2934	0.3309	0.1862	0.5881	0.0001
Amoxicillin	1.1129	0.3847	3.0432	1.4318	6.4678	0.0038
Enrofloxacin	-1.1528	0.4566	0.3157	0.129	0.7726	0.0115
Lincomycin	-1.4435	0.676	0.2361	0.0627	0.8883	0.0327
Trimethoprim*	-1.1582	0.4289	0.314	0.1354	0.728	0.0069

*in combination with sulfadimidine

S26 Table. Results of the beta linear regression model for the concentration of *C. difficile* (16s-rDNA gene) as the dependent variable in postpartum sows.

Variable	Estimate	Std.Error	p-value
Intercept	-0.195	0.3656	0.5945
Amoxicillin (oral)	0.5499	0.3169	0.0847
Enrofloxacin	1.1751	0.2491	<0.001
Florfenicol	1.0951	0.2679	<0.001

S27 Table. Results of the logistic regression model for the presence of *E. coli* F4 fimbriae (*fas*) as the dependent variable in postpartum sows.

Variable	Estimate	Std.Error	odds ratios	OR lower CI	OR upper CI	p-value
Intercept	-1.5995	0.2338	0.2019	0.1277	0.3194	0.0002
Enrofloxacin	1.0156	0.3969	2.7609	1.2682	6.0103	0.0105
Lincomycin	-1.8061	0.7849	0.2207	0.0352	0.7651	0.0214

S28 Table. Results of the beta linear regression model for the presence of *E. coli* F4 fimbriae (*fas*) as the dependent variable in postpartum sows.

Variable	Estimate	Std.Error	p-value
Intercept	3.9195	2.6215	0.1461
Enrofloxacin	-1.1526	0.5678	0.0520
Florfenicol	-1.5284	0.698	0.0370
Trimethoprim*	-0.989	0.5514	0.0837

*in combination with sulfadimidine

S29 Table. Results of the logistic regression model for the presence of *C. difficile* (16s-rDNA gene) as the dependent variable in suckling piglets.

Variable	Estimate	Std.Error	odds ratios	OR lower CI	OR upper CI	p-value
Intercept	0.489	0.1822	1.6306	1.1409	2.3306	0.0072
Benzylpenicillin*	1.6493	0.5305	5.2035	1.8394	14.72	0.0018
Tulathromycin	1.6045	0.6989	4.9755	1.2645	19.5773	0.0216

*in combination with streptomycin

S30 Table. Results of the beta linear regression model for the concentration of *C. difficile* (16s-rDNA gene) as the dependent variable in suckling piglets.

Variable	Estimate	Std.Error	p-value
Intercept	3.4447	1.7396	0.0497
Amoxicillin	0.3035	0.1563	0.0543
Ceftiofur	-0.3082	0.219	0.1617
Gentamycin	0.7732	0.3286	0.0200

S31 Table. Results of the logistic regression model for the presence of *Escherichia/Shigella/Hafnia* as the dependent variable in suckling piglets.

Variable	Estimate	Std.Error	odds ratios	OR lower CI	OR upper CI	p-value
Intercept	-8.8981	2.408	0.0002	0	0.0276	0.0004
int1	1.2784	0.2946	3.3226	1.8653	5.9185	<0.001
Amoxicillin	1.6578	0.7705	5.2475	1.159	23.7589	0.0314
Enrofloxacin	1.1468	0.9179	3.148	0.5287	19.0255	0.2115

S32 Table. Results of the beta linear regression model for the concentration of *Escherichia/Shigella/Hafnia* as the dependent variable in suckling piglets.

Variable	Estimate	Std.Error	p-value
Intercept	-8.2237	0.9944	<0.001
int1	0.7958	0.0947	<0.001
Amoxicillin	0.348	0.1384	0.0128
Colistin	-1.1254	0.3233	0.0006
Enrofloxacin	-0.4624	0.1461	0.0018
Gentamycin	-0.5051	0.2793	0.0723

S33 Table. Results of the logistic regression model for the presence of *E. coli* F6 fimbriae (*fae*) as the dependent variable in suckling piglets.

Variable	Estimate	Std.Error	odds ratios	OR lower CI	OR upper CI	p-value
Intercept	-1.5198	0.4173	0.2187	0.0965	0.4955	0.0002
Amoxicillin	1.6753	0.7876	5.3405	1.1406	25.0041	0.0334
Ceftiofur	-1.9917	0.8301	0.1364	0.0268	0.6943	0.0164
Colistin	1.6131	0.7453	5.0181	1.1645	21.6231	0.0304

S34 Table. Results of the beta linear regression model for the concentration of *C. difficile* (16s-rDNA gene) as the dependent variable in weaned piglets.

Variable	Estimate	Std.Error	p-value
Intercept	3.3895	2.0177	0.0975
Amoxicillin	-0.7446	0.2266	0.0016
Amoxicillin (oral)	0.4218	0.1945	0.0336

S35 Table. Results of the logistic regression model for the presence of *C. perfringens* (*cpa*) as the dependent variable in weaned piglets.

Variable	Estimate	Std.Error	odds ratios	OR lower CI	OR upper CI	p-value
Intercept	1.6011	0.2984	4.9583	2.7624	8.8996	<0.001
Amoxicillin (oral)	-0.8641	0.3684	0.4214	0.2047	0.8676	0.0190
Colistin (oral)	-1.1956	0.5453	0.3025	0.1038	0.8809	0.0284
Enrofloxacin	-0.9702	0.4061	0.3789	0.1709	0.84	0.0169
Oxytetracycline	-1.6011	0.6993	0.2016	0.0512	0.7942	0.0221

S36 Table. Results of the logistic regression model for the presence of *E. coli* F18 fimbriae (*fedA*) as the dependent variable in weaned piglets.

Variable	Estimate	Std.Error	odds ratios	OR lower CI	OR upper CI	p-value
Intercept	-0.7419	0.2454	0.4762	0.2944	0.7703	0.0024
Amoxicillin	-1.2955	0.3628	0.2737	0.1344	0.5574	0.0003
Doxycycline (oral)	1.3251	0.4441	3.7625	1.5757	8.984	0.0028

S37 Table. Results of the logistic regression model for the presence of *E. coli* F6 fimbriae (*fae*) as the dependent variable in weaned piglets.

Variable	Estimate	Std.Error	odds ratios	OR lower CI	OR upper CI	p-value
Intercept	-7.7639	1.3349	0.0004	0	0.0058	<0.001
int1	0.9394	0.1729	2.5585	1.8233	3.5902	<0.001
Doxycycline (oral)	0.9768	0.46	2.656	1.0781	6.5431	0.0337
Enrofloxacin (oral)	0.8049	0.4125	2.2365	0.9964	5.0199	0.0510

S38 Table. Results of the linear regression model for the concentration of *E. coli* F6 fimbriae (*fae*) as the dependent variable in weaned piglets.

Variable	Estimate	Std.Error	p-value
Intercept	-1.4074	1.3648	0.3058
int1	0.9478	0.169	<0.001
Cefquinome	0.7741	0.3965	0.0546

S39 Table. Amount of administered antibiotic agents in different farms and animal groups during the sampling period.

Farm	No. of reproductive sows	Antibiotic agent	Sows (mg)	Suckling piglets (mg)	Weaner (mg)
A	160	Amoxicillin oral	0	0	400000
		Cefquinom	0	2500	2500
		Ceftiofur	0	10000	0
		Florfenicol	30000	0	0
B	500	Amoxicillin	75000	135000	0
		Cefquinom	0	0	2500
		Enrofloxacin	10000	0	0
		Sulfonamid Trimethroprim	96000	0	0
C	140		0	0	0
D	240	Penicillin Streptomycin	0	44062	0
E	340	Amoxicillin	15000	0	0
		Amoxicillin oral	1000000	0	0
		Florfenicol	0	30000	0
		Oxytetracycline	0	40000	80000
		Penicillin Streptomycin	0	66093	0
		Sulfonamid Trimethroprim	0	24000	1440000
		Tilmicosin	0	0	3200000
		Tulathromycin	0	15000	0
		Tylosin	0	0	110000
F	350	Amoxicillin	37500	75000	81300
		Amoxicillin oral	0	0	3000000
		Doxycyclin	0	0	500000
		Enrofloxacin	0	5000	0
		Tulathromycin	0	25000	0
		Tylosin	154000	0	0
G	160	Amoxicillin	0	30000	0
		Enrofloxacin	2500	0	0
		Lincomycin	11340	0	0
H	210	Amoxicillin	0	0	900
		Amoxicillin oral	0	0	1000000
		Enrofloxacin	10000	0	0
I	450	Amoxicillin	165000	15000	25500
		Amoxicillin oral	0	0	1000000
		Sulfonamid Trimethroprim	120000	0	0

		Tulathromycin	0	15000	0
J	240	Amoxicillin	15000	90000	30000
		Amoxicillin oral	0	0	1000000
		Enrofloxacin	0	5000	5000
		Penicillin Streptomycin	60000	0	0
		Sulfonamid Trimethroprim	192000	0	0
K	200	Amoxicillin	225000	0	0
		Colistin	0	0	240000
		Florfenicol	60000	0	0
		Gentamycin	0	8500	0
		Penicillin Streptomycin	0	22031	0
L	260	Amoxicillin	0	0	15000
		Enrofloxacin	0	0	2500
		Penicillin Streptomycin	0	44062	0
		Sulfonamid Trimethroprim	24000	0	0
M	340	Amoxicillin	37500	96300	30000
		Amoxicillin oral	1000000	0	0
		Colistin	0	0	360000
		Doxycycline oral	0	0	250000
		Enrofloxacin	0	2500	0
		Lincomycin	11340	0	0
		Lincomycin oral	0	0	200000
		Sulfonamid Trimethroprim	0	0	720000
N	200	Amoxicillin	30000	30000	62100
		Enrofloxacin	12500	0	5000
		Lincomycin	11340	0	
O	120	Amoxicillin	0	0	48000
P	260	Amoxicillin	37500	0	21300
		Enrofloxacin	40000	0	2500
		Neomycin oral	1000000	0	1000000
		Tilmicosin oral	600000	0	400000
		Tulathromycin	15000	0	0
Q	280	Amoxicillin	0	30000	37500
		Ceftiofur	0	0	10000
		Colistin	0	8000	6000
		Florfenicol	30000	0	0
R	200	Amoxicillin	52500	0	0
		Amoxicillin oral	0	0	2000000
		Doxycycline oral	0	0	1500000
		Enrofloxacin	5000	15000	0
		Tildipirosin		20000	8000
S	280	Amoxicillin	120000	0	4500
		Enrofloxacin	0	5000	0
		Penicillin Streptomycin	0	22031	0
		Sulfonamid Trimethroprim	48000	0	0
T	1200	Amoxicillin	0	255000	525000
		Amoxicillin oral	0		10000000
		Enrofloxacin	0	10000	0
		Florfenicol	360000	0	0

Lincomycin	0	0	317520
Sulfonamid Trimethroprim	900000	0	0
